# LncRNA INHEG promotes glioma stem cell maintenance and tumorigenicity through regulating rRNA 2’-O-methylation

**DOI:** 10.1038/s41467-023-43113-5

**Published:** 2023-11-18

**Authors:** Lihui Liu, Ziyang Liu, Qinghua Liu, Wei Wu, Peng Lin, Xing Liu, Yuechuan Zhang, Dongpeng Wang, Briana C. Prager, Ryan C. Gimple, Jichuan Yu, Weixi Zhao, Qiulian Wu, Wei Zhang, Erzhong Wu, Xiaomin Chen, Jianjun Luo, Jeremy N. Rich, Qi Xie, Tao Jiang, Runsheng Chen

**Affiliations:** 1 Key Laboratory of Epigenetic Regulation and Intervention, Institute of Biophysics, Chinese Academy of Sciences, 100101 Beijing, China; 2 University of Chinese Academy of Sciences, 100049 Beijing, China; 3 Key Laboratory of Growth Regulation and Translational Research of Zhejiang Province, School of Life Sciences, Westlake University, 310024 Hangzhou, China; 4 Westlake Laboratory of Life Sciences and Biomedicine, 310024 Hangzhou, China; 5 Beijing Neurosurgical Institute, 100050 Beijing, China; 6 Department of Department of Orthopedics, Peking Union Medical College Hospital, 100730 Beijing, China; 7 Department of Pathology, Case Western Reserve University, Cleveland, 44106 USA; 8 Cleveland Clinic Lerner College of Medicine, Case Western Reserve University, Cleveland, 44195 USA; 9 Hillman Cancer Center and Department of Neurology, University of Pittsburgh Medical Center, Pittsburgh, 15261 USA; 10 Department of Neurosurgery, Beijing Tiantan Hospital, Capital Medical University, 100050 Beijing, China

**Keywords:** CNS cancer, Long non-coding RNAs, Cancer stem cells

## Abstract

Glioblastoma (GBM) ranks among the most lethal of human cancers, containing glioma stem cells (GSCs) that display therapeutic resistance. Here, we report that the lncRNA INHEG is highly expressed in GSCs compared to differentiated glioma cells (DGCs) and promotes GSC self-renewal and tumorigenicity through control of rRNA 2’-O-methylation. INHEG induces the interaction between SUMO2 E3 ligase TAF15 and NOP58, a core component of snoRNP that guides rRNA methylation, to regulate NOP58 sumoylation and accelerate the C/D box snoRNP assembly. INHEG activation enhances rRNA 2^’^-O-methylation, thereby increasing the expression of oncogenic proteins including EGFR, IGF1R, CDK6 and PDGFRB in glioma cells. Taken together, this study identifies a lncRNA that connects snoRNP-guided rRNA 2’-O-methylation to upregulated protein translation in GSCs, supporting an axis for potential therapeutic targeting of gliomas.

## Abstract (web summary)

Long noncoding RNAs (lncRNAs) may contribute to cancer progression. Here the authors show that LncRNA INHEG modulates rRNA 2’-O-methylation and facilitates mRNA translation to promote glioma stem cell renewal and growth.

## Introduction

Glioblastoma (GBM; World Health Organization grade IV glioma) is the most prevalent and malignant primary intrinsic brain tumor with a median survival of less than 15 months^1^. Standard-of-care for GBM includes surgical resection, concurrent radiotherapy and chemotherapy, followed by adjuvant chemotherapy, but offers only palliation. GBMs display cellular hierarchies with self-renewing glioma stem cells (GSCs) at the apex of the hierarchy^2^. GSCs are functionally defined by their capacity for self-renewal and cell differentiation and maintain tumor heterogeneity, tumor growth and therapy resistance^3^. Elucidation of molecular mechanisms regulating GSCs will expand our understanding of the disease and provide insights into effective therapeutic strategies to target GBMs.

RNA modifications represent essential mechanisms to control transcriptional regulation^4,5^. Ribose 2′-O-methylation (2′-O-Me) is the most abundant modification in human rRNA, with more than 100 sites mapped^6–8^. Human rRNA 2′-O-Me is mediated by C/D box small nucleolar ribonucleoproteins (snoRNPs) that consist of a C/D box snoRNA, the methyltransferase fibrillarin (FBL), and the RNA-binding proteins NOP58 (NOP58 Ribonucleoprotein), NOP56, and NHP2L1^9,10^. rRNA modifications play essential roles in ribosome biogenesis and translational regulation of oncogenic proteins to regulate numerous cell functions, including cell proliferation, survival, and transformation during tumorigenesis^11–13^. However, little is known about rRNA 2′-O-Me in cancer stem cells, especially GSCs.

The human genome is extensively transcribed as noncoding RNAs^14^. Long noncoding RNAs (lncRNAs) are defined as transcripts of more than 200 nucleotides that lack protein-coding potential^15^. LncRNAs play important roles in numerous diseases, ranging from influenza to acute leukemias and solid tumors^16–19^. LncRNAs modulate gene expression through transcriptional regulation, RNA turnover, and translational control^20–22^. However, the functions and modes of lncRNAs in GSCs are not well characterized.

Based on the hypothesis that rRNA methylation represents a regulatory node within the glioblastoma cellular hierarchy, we interrogated potential roles in the maintenance of GSC self-renewal. Through integrated analysis of NOP58 RNA immunoprecipitation followed by deep sequencing (RIP-seq) and the transcriptomes of human patient-derived GSCs with matched DGCs, we identify a GSC-specific lncRNA, INHEG, which interacts with NOP58 and promotes NOP58 sumoylation and downstream rRNA methylation. This process supports GSC self-renewal and tumorigenesis, offering a further understanding of rRNA methylation in cancer stem cells and providing potential therapeutic targets for lethal cancer.

## Results

### rRNA 2’-O-methylation is involved in self-renewal of GSCs

**Fig. 1:**
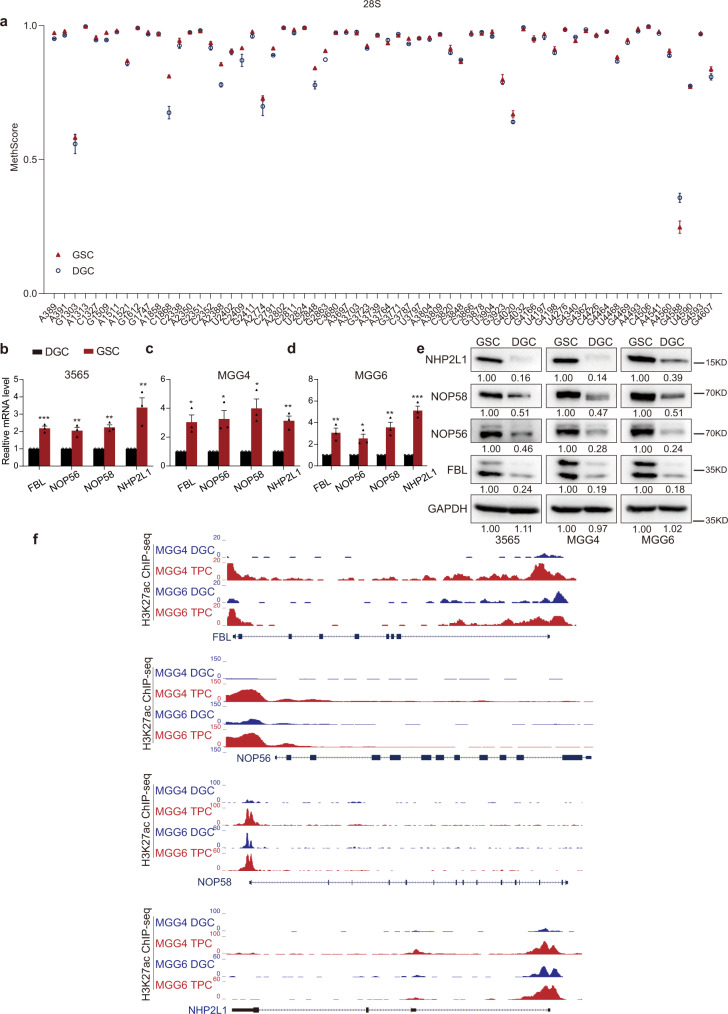
rRNA 2′-O-Me is involved in the self-renewal of GSCs. **a** MethScore values for 2′-O methylated nucleotide in 28S rRNAs (A389, A391, G1303, A1313, C1327, G1509, A1511, A1521, G1612, G1747, A1858, C1868, C2338, A2350, G2351, C2352, A2388, U2402, C2409, G2411, A2774, C2791, A2802, C2811, U2824, C2848, G2863, C3680, A3697, A3703, G3723, A3739, A3764, G3771, C3787, U3797, A3804, A3809, C3820, C3848, C3866, G3878, U3904, G3923, G4020, C4032, G4166, U4197, G4198, U4276, G4340, G4362, C4426, G4464, U4468, G4469, A4493, C4506, A4541, A4560, G4588, U4590, G4593, G4607) from patient-derived GSCs (3565 and MGG4) and matched DGCs. The MethScore is equal to the ratio of 2′-O-Me at each modified nucleotide. Data represent the mean ± SD from GSCs (3565 and MGG4) and DGCs. **b**–**d** The relative expression of FBL, NOP56, NOP58 and NHP2L1 at RNA level in patient-derived GSCs and matched DGCs, **b** for 3565, **c** for MGG4, **d** for MGG6. Data are shown as mean ± SD. *n* = 3 independent experiments. Significance determined by two-tailed Student’s *t*-test. *P < 0.05; **P < 0.01; ***P < 0.001. **e** The relative expression of FBL, NOP56, NOP58, and NHP2L1 at protein level in patient-derived GSCs and matched DGCs. Three experiments were repeated independently with similar results. **f** H3K27ac signal at the FBL, NOP56, NOP58 and NHP2L1 locus in TPCs and matched DGCs from public sequencing data (GSE54792).

As GBM represents one of the cancers from which the cellular hierarchy is most reliably derived, we leveraged primary cells obtained from freshly dissociated human glioma tissues and GSCs grown under serum-free conditions to determine the potential role of rRNA modification in cancer stem cells. Serum-induced differentiation of GSCs is associated with loss of tumorigenicity and differentiated glioma cells (DGCs) expressed low levels of the GSC marker SOX2 and BMI1 as well as high levels of astrocytes marker GFAP, which is consistent with previous studies^23,24^ (Supplementary Fig. 1a). To investigate the role of rRNA methylation in the tumor hierarchy, we measured the rRNA 2′-O-Me levels in two matched patient-derived GSCs (3565 and MGG4) and DGCs with RiboMeth-Seq^8,25,26^. The rRNA methylation ratios for multiple sites in 28S, 18S and 5.8S rRNA were decreased in DGCs compared with GSCs (Fig. 1a and Supplementary Fig. 1b, c). In addition, we measured the 28S rRNA 2′-O-Me levels in patient-derived GSCs (3565) and DGCs with RTL-P (Reverse Transcription at Low deoxyribonucleoside triphosphate (dNTP) concentration followed by polymerase chain reaction (PCR))^27^, and observed that the rRNA methylation ratios for examined sites were higher in patient-derived GSCs than in matched DGCs (Supplementary Fig. 1d). Moreover, the C/D box snoRNP components FBL, NOP56, NOP58 and NHP2L1 were also highly expressed in patient-derived GSCs (3565, MGG4, and MGG6) compared with DGCs at the mRNA level (Fig. 1b–d) and the protein level (Fig. 1e and Supplementary Fig. 1e). By in silico analysis of Histone 3 lysine 27 acetyl chromatin immunoprecipitation followed by deep sequencing (H3K27ac ChIP-seq) data from matched stem-like tumor-propagating cells (TPCs) and DGCs^23^, the C/D box snoRNP protein components (FBL, NOP56, NOP58 and NHP2L1) displayed enhanced H3K27ac signal in TPCs (Fig. 1f). Collectively, these data show that both rRNA 2′-O-Me and its molecular regulators are more abundant in GSCs compared to differentiated tumor cells.

### rRNA 2’-O-methylation regulators contribute to GSC self-renewal and growth

**Fig. 2:**
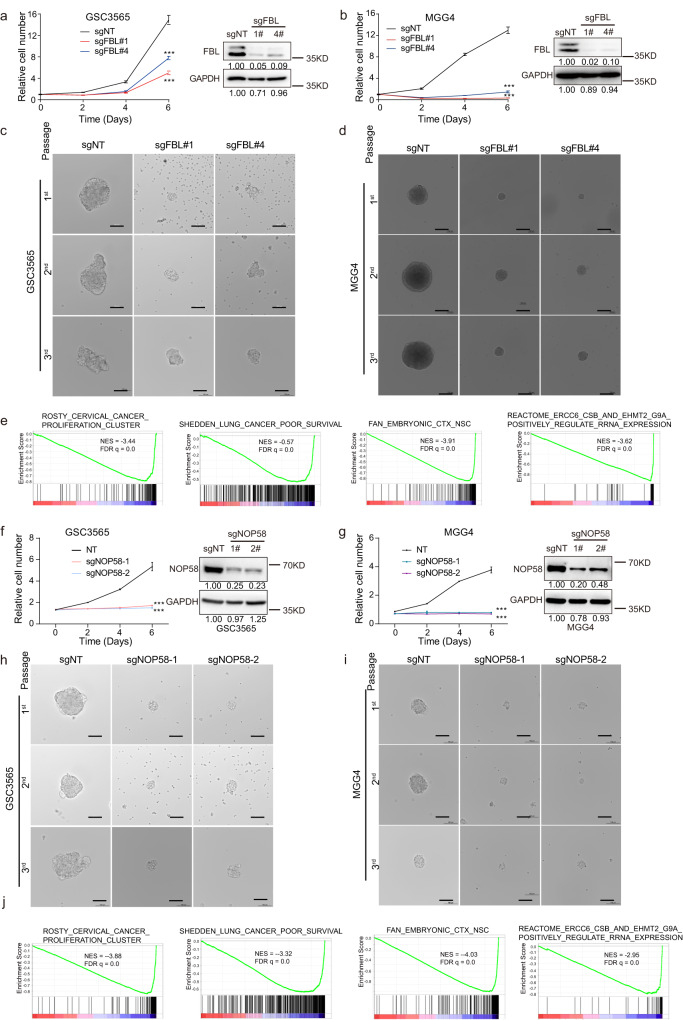
rRNA 2′-O-Me regulators are functionally critical for GSCs. **a**, **b** Relative cell number was assessed in GSC3565 (**a**, left) and MGG4 (**b**, left) cells over a 6-day time course after treatment with Cas9 and a non-targeting control or two FBL-targeting sgRNAs. Right: western blot showing the relative protein expression level of FBL. **c**, **d** Representative bright-field images from three independent experiments showing the sphere formation in GSC3565 (**c**) and MGG4 (**d**) cells after treatment with Cas9 and a non-targeting control or two FBL-targeting sgRNAs by sequential passage sphere formation assays. Scale bar, 100 μm. **e** GSEA analysis of differentially expressed genes in FBL-knocked out or control GSC3565 and MGG4 cells. NES and *q*-values were automatically determined by GSEA. **f**, **g** Relative cell number was assessed in GSC3565 (**f**, left) and MGG4 (**g**, left) cells over a 6-day time course after treatment with Cas9 and a non-targeting control or NOP58-targeting sgRNAs. Right: western blot showing the relative protein expression level of NOP58. **h**, **i** Representative bright-field images from three independent experiments showing the sphere formation in GSC3565 (**h**) and MGG4 (**i**) cells after treatment with Cas9 and a non-targeting control or two NOP58-targeting sgRNAs by sequential passage sphere formation assays. Scale bar, 100 μm. **j** GSEA analysis of differentially expressed genes in NOP58-knocked out or control GSC3565 cells. NES and *q*-values were automatically determined by GSEA. Data represent mean ± SD. *n* = 3 independent experiments. Significance determined by one-way ANOVA (**a**, **b**, **f** and **g**). *P < 0.05; **P < 0.01; ***P < 0.001.

To interrogate the roles of molecular regulators of rRNA 2′-O-Me, we knocked out FBL, the methyltransferase of rRNA 2′-O-Me, in two patient-derived GSCs using CRISPR-Cas9 technology with two distinct, non-overlapping sgRNAs. Targeting FBL expression impaired cell growth of two patient-derived GSCs (Fig. 2a, b). Moreover, low expression of FBL inhibited sphere formation not only in primary assays but also in secondary and tertiary passages (Fig. 2c, d). To interrogate the signaling pathways modulated by FBL, we performed transcriptome sequencing of 3565 and MGG4 cells with FBL knockout or non-treatment. Gene set enrichment analysis (GSEA) of differentially expressed genes demonstrated that genes regulated by FBL are related to cancer proliferation, poor survival, neural stem cells, and rRNA expression (Fig. 2e).

In orthogonal studies, we knocked out NOP58, the core RNA-binding protein of the C/D box snoRNP complex, in two patient-derived GSCs with CRISPR-Cas9 technology, decreasing cell growth and sphere formation (Fig. 2f–i). We also performed transcriptome sequencing of 3565 and MGG4 cells with NOP58 knockdown or control. GSEA of differentially expressed genes showed a similar enrichment result as FBL (Fig. 2j and Supplementary Data 1). Together, these results suggest that rRNA 2′-O-Me modification regulators promote GSC growth and self-renewal.

### LncRNA INHEG binds to NOP58 and is highly expressed in GSCs

**Fig. 3:**
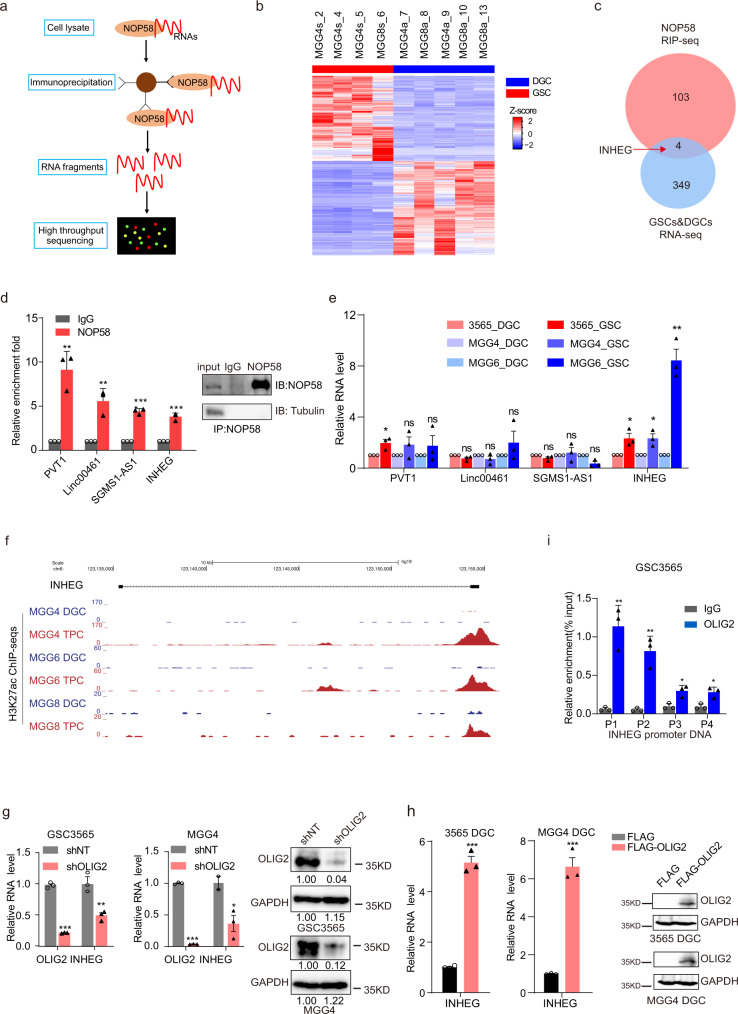
LncRNA INHEG interacts with NOP58 and is highly expressed in GSCs. **a** Workflow to generate NOP58 uvRIP-seq. **b** Cluster heatmap of 3838 differentially expressed genes between GSC and adherent cells from public data (FC > 2; FDR < 0.01). **c** Venn diagram of NOP58-enriched lncRNAs in RIP-seq and highly expressed lncRNAs in GSCs. **d** The relative enrichment fold of four NOP58-binding lncRNA candidates verified by qRT-PCR. Representative Western blot images from three independent experiments showing the efficiency and the specificity of NOP58 immunoprecipitation (right). **e** The relative expression of lncRNAs in (**d**) between patient-derived GSCs and matched DGCs detected by qRT-PCR. **f** H3K27ac signal at the INHEG locus in TPCs and matched DGCs from public sequencing data. **g** The relative expression of OLIG2 and INHEG in patient-derived GSCs treated with control or OLIG2-targeted shRNAs by qRT-PCR (left and middle). Representative Western blot images from three independent experiments showing the relative expression of OLIG2 in patient-derived GSCs treated with control or OLIG2-targeted shRNAs (right). **h** The relative expression of INHEG in differentiated glioma cells (DGCs) treated with control or OLIG2-overexpressed plasmids by qRT-PCR (left and middle). Representative Western blot images from three independent experiments showing the relative expression of OLIG2 in DGCs treated with control or OLIG2-overexpressed plasmids (right). **i** The ChIP assay of binding of OLIG2 to INHEG promoter DNA in GSC3565. P1, P2, P3, and P4 represent distinct regions of INHEG promoter. Data are shown as mean ± SD. *n*  =  3 independent experiments. Significance determined by two-tailed Student’s *t*-test. *P < 0.05; **P < 0.01; ***P < 0.001; ns, no significance.

We next sought to understand the molecular regulation of rRNA 2′-O-Me modification in GSCs. As NOP58 is proposed as an RNA-binding protein required for the accumulation of all box C/D snoRNAs^10^, we explored the RNA interactome of NOP58 using ultraviolet crosslinked immunoprecipitation followed by RNA sequencing (uvRIP-seq) (Fig. 3a and Supplementary Fig. 2a). The uvRIP-seq identified a number of NOP58-binding lncRNA candidates (Supplementary Data 2). To prioritize potential binding partners, we compared the transcriptomes of human patient-derived GSCs with DGCs from public RNA-seq data (GSE73845), revealing 3838 differentially expressed genes (protein-coding genes and lncRNAs) (Fig. 3b). Overlapping these uvRIP-seq and RNA-seq datasets, 4 lncRNAs interacted with NOP58 and were upregulated in GSCs (Fig. 3c). These four candidates were further validated to be significantly enriched with NOP58 through NOP58 RIP followed by qRT-PCR (Fig. 3d). In addition, the expression pattern of these four lncRNAs were examined in human patient-derived GSCs and matched DGCs by qRT-PCR, and the data showed that the long noncoding RNA INHEG (Interacts with NOP58 and Highly Expressed in GSCs) has higher expression in GSCs compared with matched DGCs (Fig. 3e). For the follow-up studies, we focused on an uncharacterized lncRNA, INHEG (Interacts with NOP58 and Highly Expressed in GSCs).

Analyzing publicly available sequencing data of H3K27ac ChIP from TPCs and DGCs (GSE54792) revealed that the INHEG promoter region displayed a greater H3K27ac signal in TPCs (Fig. 3f).

To delineate INHEG function further, we characterized the genomic locus on chromosome 6 containing INHEG, which also codes for FABP7 and SMPDL3A in the opposite transcriptional direction (Supplementary Fig. 2b). By 5’ and 3’ Rapid Amplification of cDNA Ends (RACE) performed in glioma cells, we discovered that INHEG consisted of two exons, containing 650 nucleotides (Supplementary Fig. 2b).

To explore the subcellular localization of INHEG, we performed RNA fluorescence in situ hybridization (RNA-FISH) in glioma cells and found that INHEG was mainly localized in the nucleus (Supplementary Fig. 2c), which was confirmed by nucleocytoplasmic fractionation followed by qRT-PCR in glioma cells and patient-derived GSCs (Supplementary Fig. 2d, e). Collectively, the lncRNA INHEG is highly expressed in GSCs and interacts with NOP58.

### OLIG2 regulates the expression of INHEG

In parallel to induced pluripotency, glioma cells can be induced into a stem-like state through overexpression of key transcription factors POU3F2, SOX2, SALL2, and OLIG2, which are required for maintaining stem-like states^23^. In this dataset, we found that INHEG was upregulated upon acquisition of the stem cell state, and its promoter region was bound by OLIG2^28^ (Supplementary Fig. 3a). Consistent with prior reports, GSCs expressed higher levels of OLIG2 than DGCs (Supplementary Fig. 3b). Based on these results, we reasoned that OLIG2 may regulate the expression of INHEG. We knocked down OLIG2 with specific targeting short hairpin RNAs (shRNAs) or siRNAs in patient-derived GSCs and glioma cells respectively, then found that INHEG expression was decreased upon OLIG2 downregulation as measured by qRT-PCR and western blotting (Fig. 3g and Supplementary Fig. 3c). Reciprocally, overexpressing OLIG2 induced INHEG in patient-derived GSCs and glioma cells (Fig. 3h and Supplementary Fig. 3d). To directly connect transcriptional control, OLIG2 ChIP followed by qPCR indicated that OLIG2 bound the promoter DNA of INHEG with normal IgG as control (Fig. 3i and Supplementary Fig. 3e, f). Furthermore, we discovered a consensus OLIG2 binding site in the promoter region of INHEG by FIMO searching^29^ (Supplementary Fig. 3g). We performed the reporter assay and found that OLIG2 regulates INHEG at the transcription level, and mutation of the OLIG2 binding site abolished the regulation of INHEG expression (Supplementary Fig. 3h). Collectively, these data indicate that OLIG2 regulates the transcription of INHEG.

### INHEG is required for GSC proliferation and self-renewal

**Fig. 4:**
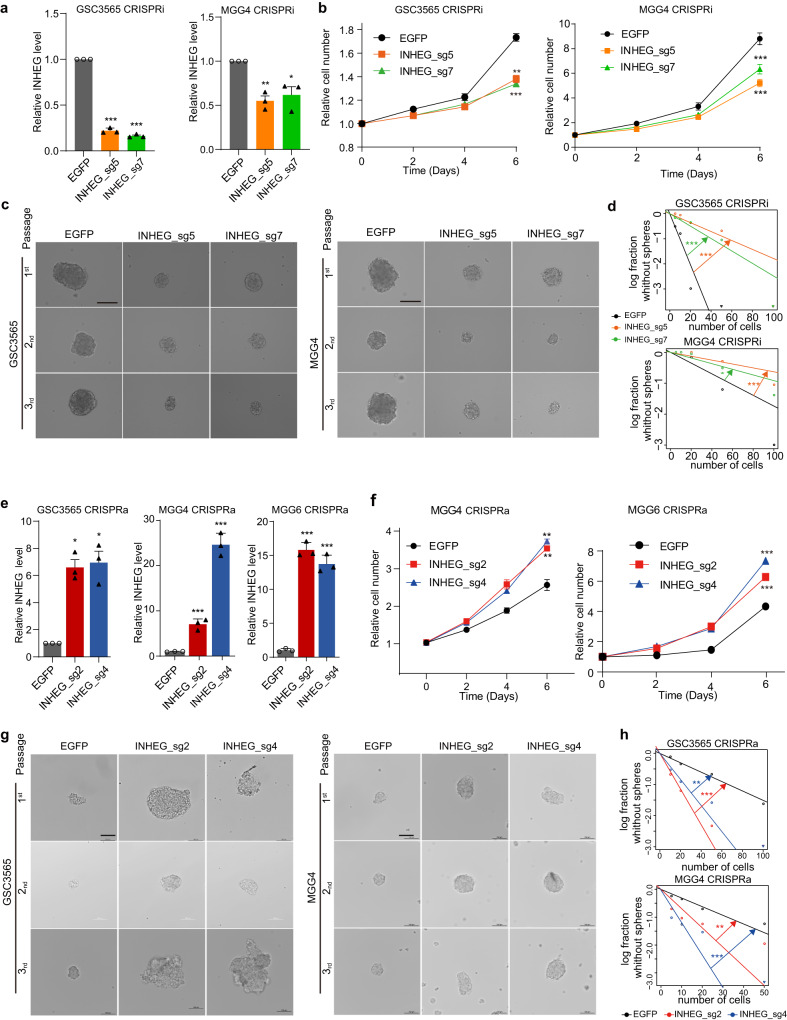
INHEG promotes self-renewal of GSCs and gliomagenesis. **a** The qRT-PCR analysis of INHEG in GSC3565 and MGG4 stably transfected with KRAB-dCas9-expressed and sgRNA-expressed plasmids. EGFP as a non-targeting control sgRNA, INHEG-sg5 and INHEG-sg7 as INHEG-targeting sgRNAs. *n* = 3 independent experiments. **b** Relative cell number of GSC3565 and MGG4 following KRAB-dCas9 mediated INHEG knockdown or treatment with a non-targeting sgRNA. Three (MGG4) or five (GSC3565) biological replicates were used for each condition. **c** Representative bright-field images from three independent experiments showing the sphere formation ability of GSC3565 and MGG4 following KRAB-dCas9 mediated INHEG knockdown or treatment with a non-targeting sgRNA were examined by sequential passage sphere formation assays. Scale bar: 150 μm. **d** Extreme limiting dilution analysis (ELDA) for sphere formation of GSC3565 (top) and MGG4 (bottom) cells following KRAB-dCas9 mediated INHEG knockdown or treatment with a non-targeting sgRNA. Three experiments were repeated independently showing similar results. **e** The qRT-PCR analysis of INHEG in GSC3565, MGG4, and MGG6 stably transfected with dCas9-VP64-expressed and sgRNA-expressed plasmids. EGFP as a non-targeting control sgRNA, INHEG-sg2 and INHEG-sg4 as INHEG-targeting sgRNAs. *n* = 3 independent experiments. **f** Relative cell number of MGG4 and MGG6 cells following dCas9-VP64 mediated INHEG overexpression or treatment with a non-targeting sgRNA. Three biological replicates were used for each condition. **g** Representative bright-field images from three independent experiments showing the sphere formation ability of GSC3565, MGG4 following dCas9-VP64 mediated INHEG overexpression or treatment with a non-targeting sgRNA were examined by sequential passage sphere formation assays. Scale bar: 100 μm. **h** Extreme limiting dilution analysis (ELDA) for sphere formation of GSC3565 (top) and MGG4 (bottom) cells following dCas9-VP64 mediated INHEG overexpression or treatment with a non-targeting sgRNA. The experiment was repeated four times independently. **a**, **e** Data are shown as mean ± SD. Significance determined by two-tailed Student’s *t*-test. **b**, **f** Data represent mean ± SD. Statistical significance was assessed using an ordinary one-way ANOVA with Dunnett multiple test correction. **d**, **h** Significance determined by pairwise Chi-square test. *P < 0.05; **P < 0.01; ***P < 0.001.

To further understand the cellular function of INHEG in GSC self-renewal maintenance, we silenced INHEG at the transcriptional level using CRISPR interference (CRISPRi) with two lentivirus-mediated small guide RNAs (sgRNAs) in patient-derived GSCs (Fig. 4a). By measuring cell viability and EDU labeling ratio, it was observed that INHEG silencing impaired GSC proliferation (Fig. 4b and Supplementary Fig. 4a, b). In addition to its role in proliferation, targeting INHEG by CRISPRi sgRNAs reduced the tumorsphere formation, as assessed by sequential passage sphere formation assays and in vitro limiting dilution assay, indicating a role in self-renewal (Fig. 4c, d).

In reciprocal gain-of-function, we activated INHEG transcription by CRISPR activation (CRISPRa) and established INHEG stably overexpressing GSCs (Fig. 4e), which promoted cell proliferation of GSCs (Fig. 4f and Supplementary Fig. 4c, d). INHEG-activated GSCs displayed increased tumorsphere formation compared to EGFP-transfected cells (Fig. 4g, h). Collectively, these findings suggest that INHEG facilitates GSC proliferation and self-renewal.

### INHEG promotes the formation of the rRNA 2’-O-methylation complex, 2’-O-methylation of rRNA, and the translation of multiple oncogenes

**Fig. 5:**
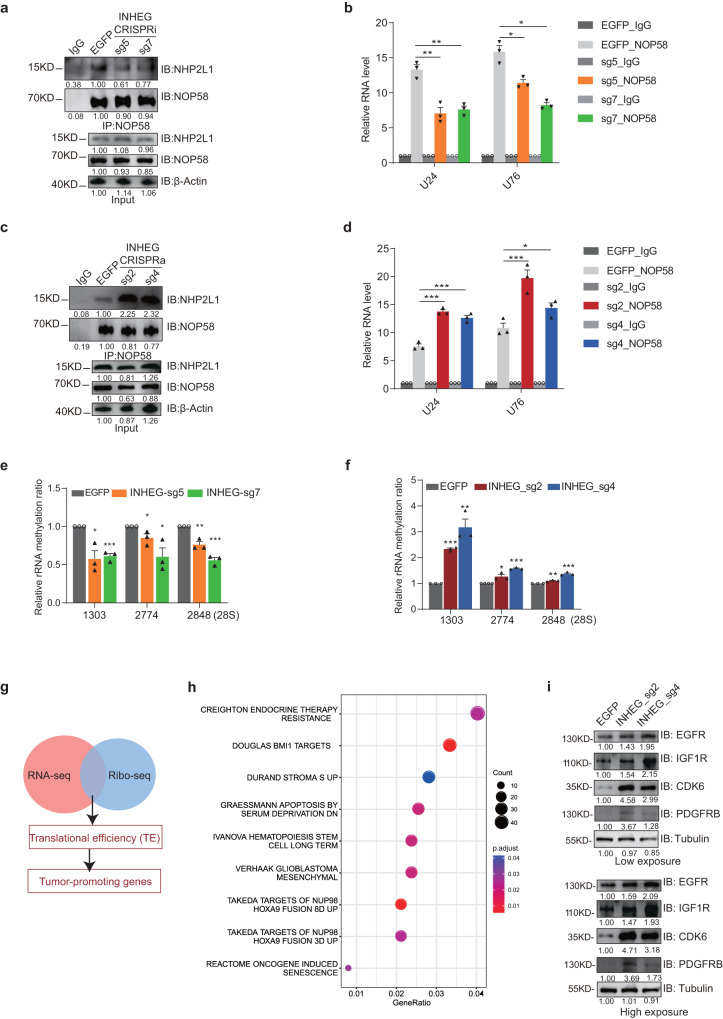
INHEG promotes rRNA 2’-O-methylating complex assembly, 2’-O-methylation of rRNA and de novo protein synthesis. **a** Co-immunoprecipitation assay of the binding of NHP2L1 to NOP58 with anti-NOP58 antibody in whole-cell lysates of U251-MG following KRAB-dCas9 mediated INHEG knockdown or treatment with a non-targeting sgRNA. **b** The relative enrichment fold of NOP58-binding snoRNAs U24 or U76 detected by qRT-PCR in U251-MG cells following KRAB-dCas9 mediated INHEG knockdown or treatment with a non-targeting sgRNA. **c** Co-immunoprecipitation assay of the binding of NHP2L1 to NOP58 with anti-NOP58 antibody in whole-cell lysates of U251-MG following dCas9-VP64 mediated INHEG overexpression or treatment with a non-targeting sgRNA. **d** The relative enrichment fold of NOP58-binding snoRNAs U24 or U76 detected by qRT-PCR in U251-MG cells following dCas9-VP64 mediated INHEG overexpression or treatment with a non-targeting sgRNA. **e** rRNA methylation ratio for sites along 28S rRNA in GSC3565 cells following KRAB-dCas9 mediated INHEG knockdown or treatment with a non-targeting sgRNA. **f** rRNA methylation ratio for sites along 28S rRNA in GSC3565 following dCas9-VP64 mediated INHEG overexpression or treatment with a non-targeting sgRNA. **g** Venn diagram showing overlaps between gene sets analyzed by RNA-seq and Ribo-seq. **h** Gene Ontology (GO) analysis of genes whose translational efficiencies were enhanced by INHEG upregulation. **i** The expression of EGFR, IGF1R, CDK6 and PDGFRB at protein level in GSC3565 cells following dCas9-VP64 mediated INHEG overexpression or treatment with a non-targeting sgRNA. Data are shown as mean ± SD. *n* = 3 independent experiments. Significance determined by two-tailed Student’s *t*-test. *P < 0.05; **P < 0.01; ***P < 0.001; ns, no significance. **a**, **c**, and **i** Three experiments were repeated independently with similar results.

C/D box snoRNPs are involved in the process of producing the site-specific ribose 2′-O-methylation. The assembly of snoRNPs involves the temporal interaction of four core proteins^30^. As one of four core members in these snoRNPs, NOP58 directly influences C/D box snoRNP biogenesis and regulates rRNA 2′-O-methylation^31,32^. The observation of interactions between INHEG and NOP58 suggests that INHEG may regulate snoRNP assembly and function. To dissect the role of INHEG in C/D box snoRNP biogenesis, we performed NOP58 immunoprecipitation in INHEG down- and upregulated glioma cells. Transcriptional inactivation of INHEG weakened the association between NOP58 and NH2PL1 that is one of box C/D snoRNPs components responsible for directly binding to snoRNAs with the expression of NOP58 and NHP2L1 unaffected (Fig. 5a). In the setting of INHEG knockdown, NOP58 pulled down less snoRNAs while the expression levels of snoRNAs remained unaltered by INHEG knockdown (Fig. 5b and Supplementary Fig. 5a). Additionally, overexpression of INHEG increased the efficiency of NOP58 interacting with NH2PL1 and snoRNAs, while NOP58, NHP2L1, and snoRNA expression remained unaltered (Fig. 5c, d, and Supplementary Fig. 5b). Our data indicate that INHEG promotes the formation of C/D box snoRNPs.

We then investigated the level of rRNA 2’-O-methylation at several sites along with 28S rRNAs by RTL-P^27^. The methylation ratios across these sites were decreased in INHEG-silenced cells compared with control cells (Fig. 5e), and similar results were observed in NOP58-deleted cells (Supplementary Fig. 5c). After overexpressing INHEG, the methylation levels of detected sites were increased (Fig. 5f).

To evaluate whether INHEG expression affects protein synthesis, RNA-seq and Ribosome profiling (Ribo-seq) were performed in control and CRISPRa-mediated INHEG overexpressed GSCs. By joint analysis of the gene transcriptional expression and ribosome protected fragments abundance, it was observed that upregulating INHEG increased the translational efficiency of more than 1000 genes (Fig. 5g). To identify signaling pathways perturbed by INHEG upregulation, we conducted gene ontology (GO) analysis and the result demonstrated that the genes with increased translation efficiency after INHEG overexpression were involved in tumorigenesis and stem cell properties (Fig. 5h). Further validation revealed that INHEG activation enhanced protein expression of oncogenes IGF1R, EGFR, CDK6, and PDGFRB while mRNA levels were unaltered, and INHEG activation led to an increased translation efficiency for IGF1R mRNA (Fig. 5i and Supplementary Fig. 5g, h), suggesting that INHEG may promote translational process of oncogenes during gliomagenesis. Thus, INHEG modulates protein synthesis of multiple oncogenes by regulating C/D box snoRNPs assembly and rRNA 2’-O-methylation status.

### INHEG-associated protein TAF15 interacts with NOP58 and functions as a SUMO2 E3 ligase for NOP58

**Fig. 6:**
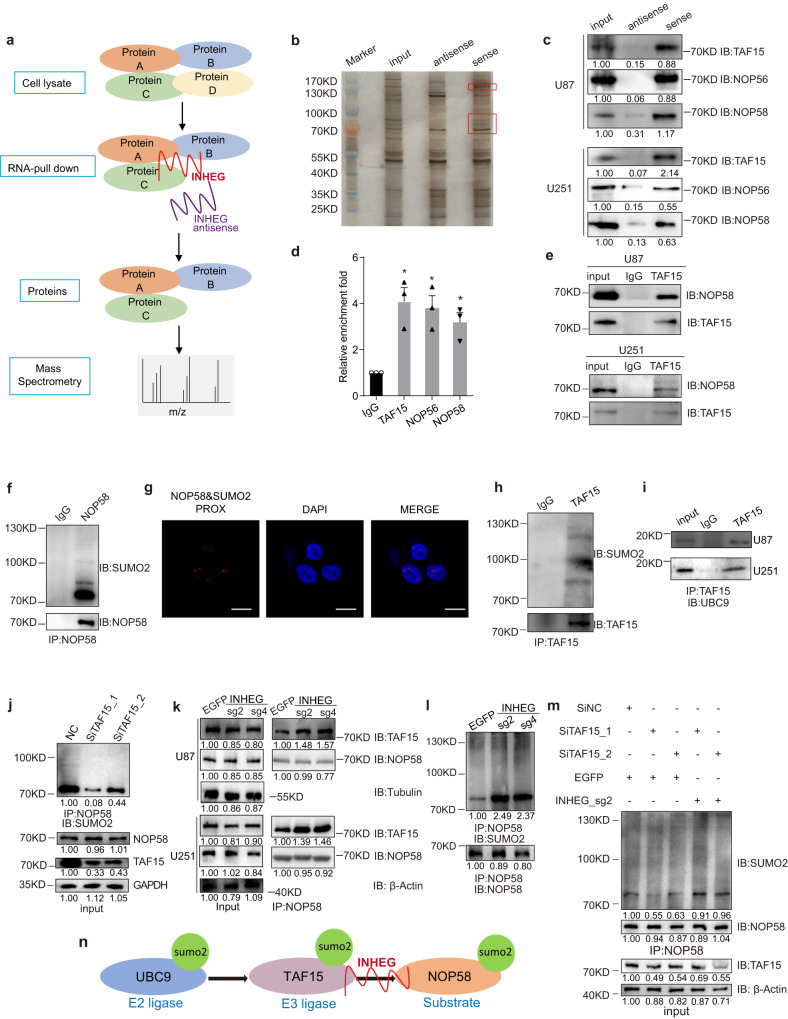
INHEG enhances the interaction between TAF15 and NOP58 and regulates NOP58 sumoylation in a TAF15-dependent way. **a** Workflow of INHEG RNA pull-down/MS assay. **b** INHEG-interacting proteins identified by RNA pull-down assay followed by silver staining. The parts in the red box and matched bands in the antisense group were analyzed by mass spectrometry. **c** INHEG-interacting proteins verified by RNA pull-down assay followed by western blot with U87-MG (top) and U251-MG (bottom) cell lysates. **d** Relative enrichment folds of INHEG by TAF15, NOP56 or NOP58 antibody in RNA-IP. Data are shown as mean ± SD. *n* = 3 independent experiments. Significance determined by two-tailed Student’s *t*-test. *P < 0.05; **P < 0.01; ***P < 0.001. **e** Co-immunoprecipitation assay of the binding of NOP58 to TAF15 with TAF15 antibody in whole-cell lysates of U87-MG and U251-MG cells. **f** Endogeneous NOP58 sumoylation detection by NOP58 immunoprecipitation and Sumo2 immunoblot with U87-MG cell lysate. **g** Representative fluorescence images from three independent proximity ligation assays showing the SUMO2 modification of NOP58 in U87-MG cell. Scale bar, 10 μm. **h** Endogeneous TAF15 sumoylation detection by TAF15 immunoprecipitation and SUMO2 immunoblot with U251-MG cell lysate. **i** Co-immunoprecipitation assay of the binding of UBC9 to TAF15 with TAF15 antibody in whole-cell lysates of U87-MG and U251-MG cells. **j** Endogeneous NOP58 sumoylation detection in U87-MG cells treated with control or TAF15-targeted siRNA. **k** Co-immunoprecipitation assay of the binding of NOP58 to TAF15 with TAF15 antibody in whole-cell lysates of U87-MG and U251-MG cells following dCas9-VP64 mediated INHEG overexpression or treatment with a non-targeting sgRNA. **l** Endogeneous NOP58 sumoylation detection in U251-MG cells following dCas9-VP64 mediated INHEG overexpression or treatment with a non-targeting sgRNA. **m** Endogeneous NOP58 sumoylation detection in U87-MG cells treated with control or TAF15-targeted siRNAs followed by dCas9-VP64 mediated INHEG overexpression or not. **n** Working model of INHEG promoting TAF15/NOP58 interaction and NOP58 sumoylation. The western blotting data show representative images from three independent experiments.

To gain insight into the mechanism of INHEG-promoted rRNA 2’-O-methylation, GSCs self-renewal and proliferation, we constructed biotin-labeled probes and performed RNA pull-down followed by mass spectrometry (MS) (Fig. 6a). Three proteins (TAF15, NOP56, and NOP58) were identified as highly enriched in the INHEG-sense condition relative to the antisense one (Fig. 6b and Supplementary Table 1). As a positive control, NOP58 confirmed the reliability of the pull-down/MS assay. In addition, immunoblots following pull-down and qRT-PCR following reciprocal immunoprecipitation confirmed interactions between INHEG with TAF15, NOP56, and NOP58 (Fig. 6c, d, and Supplementary Fig. 6a). In vitro RNA-protein binding assay with biotin-labeled INHEG and prokaryotically expressed GST-tagged proteins followed by western blot revealed that biotin-labeled INHEG pulled down GST-tagged TAF15, NOP56, and NOP58 but not GST protein, suggesting that these three proteins directly interacted with INHEG (Supplementary Fig. 6b). The association between INHEG and either TAF15 or NOP58 was further validated by Electrophoretic Mobility Shift Assay (EMSA) with prokaryotically expressed recombinant human proteins and biotin-labeled probes (Supplementary Fig. 6c). Next, to determine which region of INHEG is responsible for interacting with TAF15 and/or NOP58, we truncated INHEG into 3 fragments and performed RNA pull down, showing that the P2 (230-457 nucleotide region of INHEG) is the main fragment that binds TAF15 and NOP58 (Supplementary Fig. 6d). Additionally, we constructed truncated NOP58 regions to determine its binding site with INHEG. RNA pull-down assay demonstrated that the N-terminal domain (1-167 amino acid) of NOP58 is required for the association with INHEG (Supplementary Fig. 6e). Taken together, our data demonstrates that INHEG interacts directly with TAF15 and NOP58.

As INHEG interacts with both TAF15 and NOP58, we postulated that TAF15 might bind to NOP58 directly. To verify our hypothesis, we immunoprecipitated endogenous NOP58 and performed immunoblotting with a TAF15 antibody in glioma cells. The observation suggested that TAF15 interacted with NOP58 (Fig. 6e). Co-immunoprecipitation between TAF15 and NOP58 after ectopic expression of these two proteins in 293T confirmed the interaction (Supplementary Fig. 6f). Prokaryotically expressed recombinant human GST-tagged TAF15 and His-tagged NOP58 were generated and subjected to GST pull-down assay, revealing that GST-TAF15 interacted with NOP58 directly while the GST control failed to bind NOP58 (Supplementary Fig. 6g).

NOP58 is sumoylated in U2OS cells, and this post-translational modification is important for high-affinity snoRNA binding to form C/D box snoRNP complex^32^. To investigate NOP58 sumoylation in glioma cells, we immunoprecipitated endogenous NOP58 then performed immunoblotting with small ubiquitin-related modifier 2 (SUMO2) antibody, revealing that endogenous NOP58 was sumoylated by SUMO2 (Fig. 6f). In addition, proximity ligation assay (PLA) validated the specificity of NOP58 sumoylation (Fig. 6g). Transfection of 293T cells with GFP-tagged NOP58 and His-tagged SUMO2 plasmids followed by GFP co-immunoprecipitation revealed that NOP58 was exogenously modified by SUMO2 (Supplementary Fig. 6h). These results suggest that NOP58 is modified by SUMO2 in glioma cells.

The FET family of RNA-binding proteins includes FUS, EWSR1, and TAF15, which possess similar domain organization and biological functions^33,34^. Moreover, FUS sumoylates Ebp1 as a SUMO2 E3 ligase^35^. The association of TAF15 and NOP58 prompted us to investigate whether TAF15 is a SUMO2 E3 ligase for NOP58. We performed endogenous immunoprecipitation with TAF15 antibody that displayed a larger band in western blot, suggesting endogenous TAF15 sumoylation (Fig. 6h). We then immunoprecipitated TAF15 and detected SUMO2 E2 ligase UBC9 and observed that TAF15 interacted with UBC9 (Fig. 6i). To explore the function of TAF15 in NOP58 sumoylation, FLAG-UBC9 or FLAG-TAF15 was transfected in glioma cells (Supplementary Fig. 6i). NOP58 sumoylation increased with overexpression of either TAF15 or UBC9 (Supplementary Fig. 6j). Immunoprecipitation of endogenous NOP58 followed by immunoblotting with SUMO2 antibody in TAF15-knockdown cells and control cells indicated that endogenous NOP58 sumoylation was reduced after treating glioma cells with TAF15-targeted siRNAs (Fig. 6j and Supplementary Fig. 6k). These data indicate that TAF15 acts as a SUMO2 E3 ligase and mediates NOP58 sumoylation by recruiting the SUMO2 E2 ligase (UBC9).

### INHEG enhances the interaction between TAF15 and NOP58 and regulates NOP58 sumoylation

To explore the effect of INHEG on the association between TAF15 and NOP58, we performed endogenous co-immunoprecipitation of TAF15 and NOP58 in INHEG transcriptionally activated glioma cells. The interaction between TAF15 and NOP58 increased in INHEG-activated cells compared to cells expressing EGFP sgRNA (Fig. 6k and Supplementary Fig. 7a). Likewise, INHEG overexpression led to enhanced TAF15-NOP58 association upon exogenous co-immunoprecipitation of TAF15 and NOP58 after ectopic expression of INHEG (Supplementary Fig. 7b). Conversely, the TAF15-NOP58 association was weakened after treating cell lysates with RNase A (Supplementary Fig. 7b). In vitro transcribed INHEG facilitated the interaction between GST-TAF15 and His-NOP58 (Supplementary Fig. 7c). Collectively, these data indicate that INHEG enhances the interaction between TAF15 and NOP58.

Supporting INHEG regulation of NOP58 sumoylation, we found that inactivation of INHEG significantly weakened the SUMO modification of NOP58 (Supplementary Fig. 7d). Further, endogenous NOP58 sumoylation was detected in INHEG-activated glioma cells, and upregulation of INHEG increased NOP58 sumoylation (Fig. 6l). Moreover, we upregulated the expression of INHEG in TAF15-knockdown cells, finding that decreased NOP58 SUMO2 modification was rescued by activation of INHEG (Fig. 6m and Supplementary Fig. 7e). Taken together, these results suggest that lncRNA INHEG enhances the TAF15-NOP58 interaction by binding both NOP58 and SUMO2 E3 ligase TAF15, leading to NOP58 sumoylation (Fig. 6n).

### Regulators of rRNA 2’-O-Me promote gliomagenesis

**Fig. 7:**
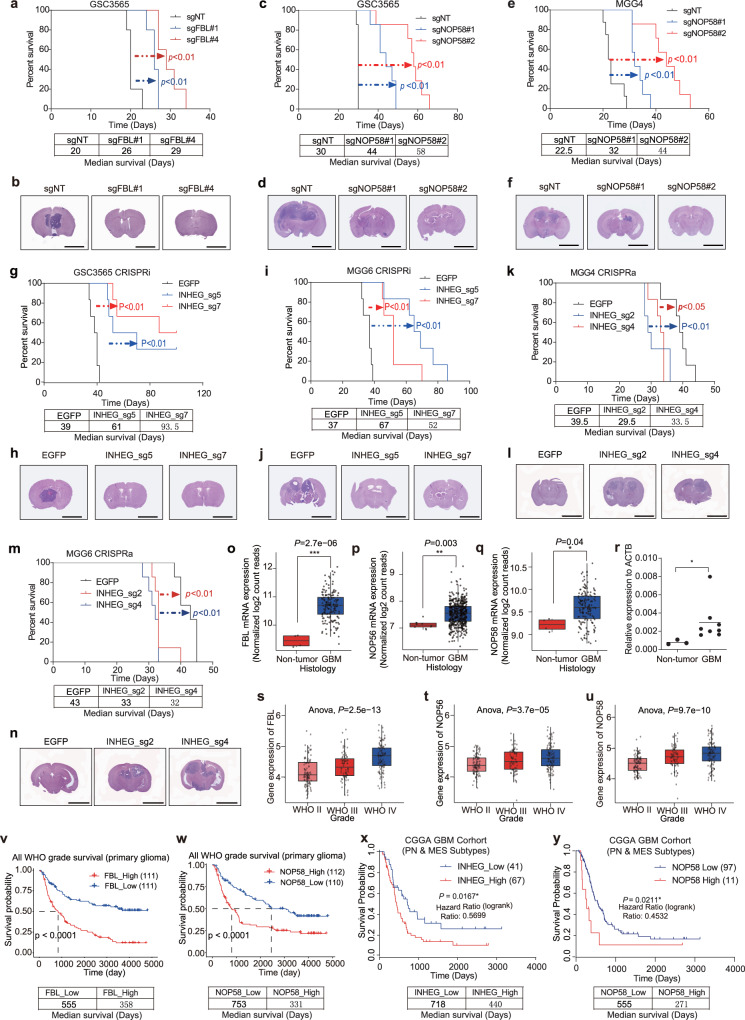
Contribution of FBL, NOP56, NOP58, and INHEG in gliomagenesis. **a** Kaplan–Meier curve showing survival of mice implanted with control or FBL-knockout GSC3565 cells. **b** Hematoxylin and eosin (HE) staining of brains implanted with cells as (**a**). Scale bar, 5 mm. **c**, **e** Kaplan–Meier curve showing survival of mice implanted with control or NOP58-knockout GSC3565(c) or MGG4(e) cells. **d**, **f** HE staining of brains implanted with cells as (**c**) or (**e**). Scale bar, 5 mm. **g**, **i** Kaplan–Meier curve showing survival of NSG mice implanted with control or INHEG-inactivated GSC3565 (**g**) or MGG6 (**i**) cells. **h**, **j** HE staining of brains implanted with cells as (**g** or **i**). Scale bar, 5 mm. **k**, **m** Kaplan–Meier curve showing survival of NSG mice implanted with control or INHEG-activated MGG4 (**k**) or MGG6 (**m**) cells. **l**, **n** HE staining of brains implantation with cells as (**k** or **m**). Scale bar, 5 mm. **o**–**q** Analysis of transcriptome data from TCGA for the mRNA level of FBL (**o**), NOP56 (**p**), and NOP58 (**q**) in non-tumor brain compared with glioblastoma. *n* = 4 vs. 156 (**o**), 10 vs. 528 (**p**), 4 vs. 156 (**q**). **r** The expression of INHEG in human normal brain tissue (*n* = 3) and glioma tissue samples (*n* = 8) examined by qRT-PCR. **s**–**u** Analysis of transcriptome data from CGGA for the mRNA level of FBL (**s**), NOP56 (**t**), and NOP58 (**u**) in high-grade glioma compared with lower-grade glioma. *n* = 194, 85, and 145 for WHO II, III, and IV, respectively. **v**, **w** Analysis of correlation between FBL (**v**) or NOP58 (**w**) mRNA expression and glioma patient survival in CGGA cohort. **x**, **y** Kaplan–Meier curve showing overall survival of GBM patients (PN and MES subtypes) divided based on INHEG (**x**) or NOP58 (**y**) expression. **a**, **c**, **e**, **g**, **i**, **k** and **m**. Data are shown as mean ± SD. *n* = 5 mice per group. Log-rank analysis was used to assess significance. **b**, **d**, **f**, **h**, **j**, **l**, and **n** Representative images from 3 mice. **o**–**q** *P*-values are calculated using Tukey’s honestly significant difference test (95% confidence interval, two-sided). **r** *P*-values are calculated using Student’s *t*-test. **s**–**u** *P*-values are calculated using one-way ANOVA test. In each box plot, the center line indicates the median, the edges of the box represent the 25th and 75th percentile, and the whiskers extend to span a 1.5 interquartile range from the edges. **v**–**y** *P*-values were derived by two-sided log-rank test. *P < 0.05; **P < 0.01; ***P < 0.001.

To explore the function of rRNA 2’-O-Me regulators on in vivo tumor growth, we generated orthotopic xenografts and observed a longer survival time and reduced tumor size in mice bearing tumors derived from FBL or NOP58-knockout GSCs compared to GSCs transduced with a control sgRNA (Fig. 7a–f). Meanwhile, we established orthotopic xenografts with patient-derived GSCs transduced with either control or INHEG-targeting sgRNAs and observed a longer survival time and reduced tumor mass in mice bearing INHEG-depleted GSCs than those bearing control GSCs (Fig. 7g–j). Mice bearing orthotopic INHEG-activated patient-derived GSCs displayed shorter survival than those bearing control GSCs (Fig. 7k, m) and had larger tumor masses on histologic analysis (Fig. 7l, n).

To explore the potential clinical significance of the genes involved in rRNA 2’-O-Me, we performed in silico analysis of these genes in the glioma tissue database. Glioblastoma frequently expressed higher levels of FBL, NOP56, and NOP58 compared to non-tumor specimens in The Cancer Genome Atlas (TCGA) RNA-seq data (Fig. 7o–q). The expression detection of INHEG by qRT-PCR displayed that INHEG was highly expressed in glioma compared to non-tumor brain tissues (Fig. 7r). FBL, NOP56, and NOP58 expression increased with tumor grade in the Chinese Glioma Genome Atlas (CGGA) (Fig. 7s–u), and FBL/NOP58 were associated with poor patient survival (Fig. 7v, w). Glioblastomas have been classified by transcriptional signatures with a transition between a proneural (PN) to mesenchymal (MES) state associated with resistance to radiation^36–38^. Therefore, we evaluated GBM patients diagnosed with either PN or MES subtype who received chemoradiation after tumor resection in the CGGA, revealing that high INHEG or NOP58 levels portended a shorter survival within the PN and MES subgroup (Fig. 7x, y). Furthermore, we analyzed publicly available single-cell RNA sequencing (scRNA-seq) data (GSE131928)^39^, and the result showed that rRNA 2’-O-methylation-associated proteins, NOP58 and FBL, were significantly co-expressed with glioma stem cell markers, SOX2 and OLIG2 (Supplementary Fig. 8a–d). Collectively, these data suggest that the pathway responsible for rRNA 2′-O-Me plays a critical role in patient outcome.

## Discussion

The ribosome is a fundamental and ubiquitous cellular engine, and 2′-O-methylation of ribosomal RNA modulates the assembly of functional ribosomes and the efficiency of translation^40–42^. 2’-O-methylation modification contributes to ribosome heterogeneity and is dysregulated in human diseases^25^. rRNA 2’-O-Me is linked to the etiology of dyskeratosis congenita and is crucial for the maintenance of hematopoietic stem cells^43^. In breast cancers, high expression of rRNA methyltransferase FBL accompanied by 2’-O-Me pattern modification is involved in impaired translational fidelity and downstream tumorigenesis^12^. Increased methylation ratios of several sites on the 28S and 18S rRNAs are associated with more aggressive breast cancer cell lines^11^. The induction of C/D box snoRNPs with the increased rRNA 2’-O-Me is required for leukemic stem cell self-renewal^44^. Our results complement these findings by suggesting that rRNA methylation is associated with glioma proliferation and GSC maintenance. Compared to differentiated glioma cells, GSCs exhibit high expression levels of components in C/D box snoRNP and elevated rRNA methylation ratios at multiple sites along with 28S rRNA, 18S rRNA or 5.8S rRNA. Inhibition of the C/D box snoRNP restrains GSC self-renewal and tumorigenesis. rRNA 2’-O-Me modification regulates the translation of key regulators responsible for GSC self-renewal. Our results suggest the 2’-O-Me modification as a critical factor in glioma stem cell biology and uncover this pathway dysregulation in glioblastoma.

LncRNAs are involved in multifaceted biological processes, including cell fate decision, antiviral innate immune, and tumorigenesis among others^18,45–48^. Nevertheless, lncRNAs contributions to glioma biology remain poorly understood. Here, we identified a key lncRNA by comparing the transcriptomes of human patient-derived GSCs with matched DGCs. LncRNAs function through various modes of action, ranging from DNA replication, RNA transcription, protein translation to post-translational modification^22,49–51^. However, lncRNAs playing roles in the modulation of rRNA methylation is largely unknown. The RNA-binding protein NOP58 is the core protein of the snoRNP that is important for self-renewal of GSCs. A previous study identified NOP58-binding snoRNAs^52^. To explore other types of RNA, including lncRNAs, to which NOP58 binds, we performed NOP58 immunoprecipitation followed by RNA-seq. The intersection of lncRNAs highly expressed in GSCs and enriched by NOP58 antibody was identified. Among the four lncRNAs, Linc00461 and PVT1 are previously reported to promote the progression of glioma^53–55^. One of the intersecting lncRNAs, an uncharacterized transcript, INHEG, is distinguished by its pleiotropic roles both in GSC self-renewal and rRNA 2’-O-Me. High expression of INHEG increases the ratio of 2’-O-Me for sites along ribosomal RNA at post-transcriptional level and facilitates mRNA translation to maintain the self-renewal of GSCs. Hence, lncRNA INHEG may provide an expanded understanding of GSCs, by regulating the 2’-O-Me level of rRNA.

We identified NOP58 as the effector through which INHEG acts as a reinforcer to promote rRNA modification. As a core protein of C/D box snoRNP, NOP58 along with other components are responsible for ribose methylation of rRNA^9,10^. NOP58 is highly expressed in several cancer types and is associated with poor survival^56^. Here, we demonstrate that NOP58 contributes to the self-renewal of GSCs and tumorigenesis. One of NOP58 post-translational modifications, sumoylation, was reported to be essential for C/D box snoRNA subcellular localization and high-affinity NOP58 binding to snoRNAs^32^. A previous study showed that SUMO E3 ligase contributes to the specificity of sumoylation by selecting the substrate^57^. The SUMO2 E3 ligase for NOP58 needs to be explored. The RNA-binding protein TAF15 has the RanBP2-type zinc finger (ZnF) domain that is important for one class of human SUMO E3 ligase^57^. Here, we found that TAF15 functions as a SUMO2 E3 ligase for NOP58. Our results showed that TAF15 is modified by SUMO2, consistent with a previous report^58^. TAF15 interacts directly with SUMO2 E2 ligase UBC9, SUMO2, and its substrate NOP58, enhancing SUMO2 modification of NOP58, which meets the criteria of SUMO E3 ligase. Therefore, our data identify TAF15 as a member of the SUMO E3 ligases family. It is possible that SUMO2 E3 ligase TAF15 together with other sumoylation-associated proteins form a sumoylation machinery to promote SUMO2 modification of NOP58. Among the machinery, the lncRNA INHEG acts to enhance NOP58-TAF15 interaction and increase NOP58 sumoylation. As a result, the lncRNA INHEG is involved in the process of C/D box snoRNP biogenesis by regulating SUMO2 modification of NOP58.

In summary, our study shows that rRNA 2′-O-Me is an essential event in the self-renewal maintenance of GSCs. A lncRNA, INHEG, is observed to not only interact with NOP58 but also be highly expressed in GSCs. INHEG promotes NOP58 sumoylation by enhancing TAF15-NOP58 interaction, along with C/D box RNP assembly, rRNA methylation and de novo protein synthesis, then downstream self-renewal of GSCs (Supplementary Fig. 8). This axis may provide treatment strategies for glioblastoma.

## Methods

### Ethics statement

All mice procedures in this study were performed under an animal protocol approved by the Institutional Animal Care and Use Committee guidelines of Westlake University. The procedures and protocols for glioma patients were approved by the institutional review board of Beijing Tiantan Hospital. Informed consent was obtained from all participants.

### Cell culture

HEK293T cells (CRL-3216), U251 and U87 glioma cells were obtained from American Type Culture Collection (ATCC). MGG4 and MGG6 GSCs lines were from the Laboratory of Hiroaki Wakimoto in Massachusetts General Hospital. GSC3565 lines were derived from human (female) primary GBM in our labs.

U87-MG (with mcherry) cells, HEK293T cells were cultured in Dulbecco’s modified Eagle’s medium (DMEM, Gibco) medium supplemented with 10% fetal bovine serum (FBS) plus antibiotics at 37 °C in a 5% CO_2_ atmosphere. U251-MG cells were cultured in DMEM/F12 medium supplemented with 10% fetal bovine serum (FBS) plus antibiotics at 37 °C in a 5% CO2 atmosphere.

All glioma stem cells (GSCs) were cultured in Neurobasal media (Gibco) supplemented with 2% B27 supplement (Gibco), 1% GlutaMax Supplement (Gibco), 1% sodium pyruvate (Gibco), 1% penicillin/streptomycin (Invitrogen), 20 ng/mL basic human fibroblast growth factor (R&D), and 20 ng/mL human epidermal growth factor (R&D). For differentiation experiments, GSCs were cultured in DMEM supplemented with 10% fetal bovine serum (Gibco), 1% penicillin/streptomycin (Gibco), and 1% GlutaMax Supplement (Gibco) to induce differentiation.

### Plasmid and lentiviral transfection

Plasmid transfection was carried out with jetPRIME (Polyplus-transfection) or Lipofectamine 3000 (Thermo Fisher) for U87-MG, U251-MG and HEK293T cells with 80–90% transfection efficiency in general.

HEK293FT cells were used to generate lentiviral particles by co-transfecting the packaging vectors psPAX2 and pMD2.G with sgRNA plasmid using polyethylenimine (PEI) transfection reagent (polyscience) following the manufacturer’s instructions. Briefly, HEK293FT cells were seeded in DMEM, high glucose in 10% FBS with 1% Penicillin-Streptomycin. Twenty hours later, sgRNA plasmids, psPAX2 and pMD2.G were combined into a tube, PEI was diluted and added followed by 15 min incubation. The transfection mixture was then added to the HEK293FT cells. The media was changed after 12 h. Lentiviral particles were collected 48 h after media change and concentrated using the lentivirus concentrated kit (Genomeditech) according to the manufacturer’s instructions. Viral supernatants were centrifuged at 1500×*g* for 45 min and viral pellets were resuspended with stem cell media and frozen at −80 °C for future use.

### RNA isolation, real-time–qPCR analysis

Total RNA or RIP RNA was extracted from cells and tissues using the Trizol total RNA isolation reagent (Invitrogen) according to the manufacturer’s protocol. Specific quantitative real-time PCR experiments were performed using the TransScript II Green One-Step qRT-PCR SuperMix (Transgen), according to the manufacturer’s instructions. The primers used for real-time–qPCR analysis are summarized in Supplementary Data 3.

### Rapid amplification of cDNA ends (RACE)

5′ and 3′ RACE were performed using the FirstChoice RLM-RACE Kit (Thermo Fisher Scientific) according to the instruction manual. The primers used are listed in Supplementary Data 3.

### rRNA methylation quantification by RTL_P

We used RTL-P to measure site-specific rRNA methylation as described previously^27^ with minor modification. RT was performed in a 25 μL reaction mixture containing 100 ng of total RNA, 1 μL (10 mM) specific RT primers and a low (0.5 μM) or high (1 mM) concentration of dNTP. The primer/RNA mixture was denatured at 70 °C for 5 min and then chilled on ice. After an initial annealing step at 42 °C for 10 min, 200U of M-MLV reverse transcriptase (Invitrogen) and 0.5U RNasin Ribonuclease Inhibitor (Promega) were added. The reaction was incubated at 37 °C for 1 h and then heated at 75 °C for 15 min to deactivate the reverse transcriptase.

### Cell viability

Cell viability experiments were performed by plating cells of interest at a density of 1500 cells per well in a 96-well plate with 5 replicates. CellTiter-Glo (Promega) was used to measure relative cell number. All data were normalized to day 0 and presented as mean ± SD.

### Sphere formation assay

Sphere formation was measured by sequential passage sphere formation and in vitro limiting dilution. Single primary spheres were dissociated and plated into the 96-well plate to form secondary sphere, and single secondary spheres were dissociated and plated into 96-well plate to form third passage sphere. For in vitro limiting dilution assay, decreasing numbers of cells per well (100, 50, 20, and 10) were plated into 96-well plates. The presence and number of spheres in each well were recorded 7 days after plating. Extreme limiting dilution analysis was conducted using software available at http://bioinf.wehi.edu.au/software/elda. All tumorsphere and proliferation experiments were performed at least 3 times.

### Edu labeling and detection

Cell productive capacity was cytochemically detected according to the manufacturer’s instructions (C0071L, Beyotime, China). Briefly, GSCs were labeled with 10uM Edu for 2 h. Then, GSCs were pipetted into a cell-concentrating apparatus mounted to a microscope slide and subsequently centrifuged such that the cells were flattened against the glass. The cells were fixed by 4% polyformaldehyde for 20 min and incubated in PBS containing 0.3% Triton X-100. The cells were stained the nuclear with DAPI. Images were captured using an LSM 700 confocal microscopy platform (Carl Zeiss, Jena, Germany).

### Dual luciferase reporter assays

For INHEG promoter luciferase assay, the wild type and mutant INHEG promoter were cloned into pPRO-RB-Report luciferase reporter vector (RIBOBIO), and 293T cells were co-transfected with pPRO-RB-Report-INHEG-WT/pPRO-RB-Report-INHEG-mut and pCMV-3×Flag-OLIG2/ pCMV-3×Flag. At 24 h after transfection, luciferase activity was measured using a Duo-Lite Luciferase Assay System (DD1205, Vazyme) according to the manufacturer’s instructions. Firefly luciferase was used as an internal control for normalization.

### RNA fluorescence in situ hybridization (RNA-FISH)

RNA-FISH was performed as described previously with minor modification^47^. Appropriate amounts of cells were seeded on glass coverslips. After adhesion, cells were fixed with 4% (w/v) paraformaldehyde in PBS. Then, samples were incubated with pre-cold permeabilization buffer (1× PBS, 0.5% (v/v) Triton X- 100) for 5 min at 4 °C. INHEG was detected with in vitro transcribed DIG-labeled RNA probe in hybridization buffer (50% (v/v) formamide, 5× SSC, 500 μg/μL yeast tRNA, 1× Denhardt’s solution, 500 μg/mL herring sperm DNA, 50 μg/mL Heparin, 2.5 mM EDTA, 0.1% (v/v) Tween-20, 0.25% (w/v) CHAPS) at 65 °C for 1 h. Coverslips with samples were washed with washing buffer (4× SSC, 0.1% (v/v) Tween-20) for five times, 2× SSC buffer once, 1× SSC buffer once, PBS buffer containing 3% (v/v) H_2_O_2_ for three times, and TN buffer (0.1 M Tris-HCl pH 7.5, 0.15 M NaCl) once. The samples were incubated for 30 min at room temperature in TNB blocking buffer (0.1 M Tris-HCl pH 7.5, 0.15 M NaCl, 0.5% (w/v) blocking reagent), and then they were incubated for 30 min again after anti-DIG antibody (Abcam, ab51949) was added to the buffer. After samples were washed with TNT buffer, TSA solution (PerkinElmer Life and Analytical Sciences) was applied onto the samples and incubated for 5–10 min at room temperature. Nuclear staining was performed with NucBlue Live ReadyProbes Reagent (Thermo Fisher Scientific) for 30 min at room temperature. Samples were mounted onto clean glass slides with antifading agents (ProLong® Gold Antifade Reagent, Thermo Fisher Scientific). The coverslips containing samples were sealed with nail polish. Images were acquired using an LSM 700 laser scanning confocal microscope (Zeiss).

### Protein expression and purification

His-tagged full-length TAF15 or NOP58 in pET-28a was transformed into E.coli expression strain BL21 (TransGene Biotech) for expression. LB culture supplemented with kanamycin was inoculated with a single colony at 200 rpm, 37 °C. After overnight growth, the culture was diluted 100-fold into 100 mL LB culture supplemented with kanamycin. When the absorbance at a wavelength of 600 nm reached 0.5, protein expression was induced by adding 0.5 mM IPTG. After overnight incubation at 200 rpm, 16 °C, cell pellets were harvested by centrifugation at 4 °C. His-tagged proteins were purified with Ni-NTA Fast Start Kit (Qiagen) according to the instruction manual. The concentration of purified protein was determined with the BCA Protein Assay Kit (Beyotime Biotechnology) and checked by SDS-PAGE.

GST-tagged full-length TAF15, NOP56, or NOP58 in pGEX-6p-1 was transformed into E.coli expression strain BL21 (TRANSGEN BIOTECH) for expression. LB culture supplemented with ampicillin was inoculated with a single colony at 200 rpm, 37 °C. After overnight growth, the culture was diluted 100-fold into 100 mL LB culture supplemented with ampicillin. When the absorbance at a wavelength of 600 nm reached 0.5, protein expression was induced by adding 1 mM IPTG. After overnight incubation at 200 rpm, 16 °C, cell pellets were harvested by centrifugation at 4 °C and resuspended in 10 mL pre-cold PBS containing 1 mM PMSF. After sonication, the solution was centrifuged at 16,000×*g* for 30 min and the supernatant lysates were incubated with Glutathione Sepharose (GE Healthcare) for 2 h at 4 °C. The GST-tagged proteins bound to the beads were pelleted at 500 g for 5 min and washed three times with 1 mL PBS buffer containing 1% Triton X-100 and then eluted with 10 mM reduced L-glutathione in 50 mM Tris-HCl, pH8.0. The concentration of purified protein was determined with the BCA Protein Assay Kit (Beyotime Biotechnology) and checked by SDS-PAGE.

### Nuclear and cytoplasmic RNA fractionation, RNA immunoprecipitation

RNA immunoprecipitation was performed as previously described^59^. In brief, U251-MG or U87-MG cells (4 × 10^7^) in the culture dish were crosslinked by UV light and collected. Subsequently, we use NE-PER™ Nuclear and Cytoplasmic Extraction Kit (Thermo Scientific) to isolate and cytoplasmic. Nuclear fractions of U251-MG or U87-MG cells were suspended in 2 mL RNA immunoprecipitation (RIP) buffer (50 mM Tris pH 7.4, 150 mM NaCl, 0.05% Igepal, 0.5% NP-40, 0.5 mM PMSF, 1×protease inhibitor cocktail (Roche) and 1000× RNasin® Ribonuclease Inhibitor (Promega)) followed by sonication. Cell lysates were centrifuged at 16,000×*g* for 15 min at 4 °C and the supernatants were precleared with 10 μL Dynabeads Protein G (Invitrogen). The precleared supernatants were then divided into two parts equally and incubated with antibodies for specific antibodies or isotype control IgG for 3–5 h at 4 °C, and then incubated with 20 µL Dynabeads Protein G (Invitrogen) for 2–4 h at 4 °C, followed by washing four times with high salt buffer (50 mM Tris pH 7.4, 300 mM NaCl, 0.05% Sodium Deoxycholate, 0.5% NP-40, 0.5 mM PMSF, 1×protease inhibitor cocktail (Roche) and 1000× RNasin® Ribonuclease Inhibitor (Promega)). The beads were incubated with elution buffer (100 mM Tris pH 6.8, 4% SDS, 10 mM EDTA, 1000×RNasin® Ribonuclease Inhibitor (Promega)) and 10 μL proteinase K (10 mg/mL) at 55 °C for 30 min. One-third of the eluted sample was used for western blot and the remainder was used for RNA extraction.

### Co-immunoprecipitation assay

Whole-cell lysates were prepared using lysis buffer (50 mM Tris pH 7.4, 150 mM NaCl, 0.05% Igepal, 0.5% NP-40, 0.5 mM PMSF, 1×protease inhibitor cocktail) and centrifuged at 15,000×*g* for 10 min at 4 °C. The supernatants were incubated with anti-NOP58, anti-TAF15 or anti-SUMO antibodies for 3-4 h at 4 °C, and the immune complexes were captured on Dynabeads Protein G beads (Invitrogen) for 2–4 h at 4 °C. Then, the co-immunoprecipitate was eluted by high salt buffer (50 mM Tris pH 7.4, 300 mM NaCl, 0.05% Sodium Deoxycholate, 0.5% NP-40, 0.5 mM PMSF, 1×protease inhibitor cocktail) and analyzed by SDS–PAGE.

### Proximity ligation assay

This assay was performed as reported^60^. For analysis of cultured cells, cells were grown on chamber slides (Nunc, #154534) for at least 16 h, washed twice with PBS, and fixed in 3.7% formaldehyde in PBS for 15 min at room temperature. Then, the slides were washed with TBS (25 mM Tris, 100 mM NaCl, pH 7.4), incubated for 10 min in 50 mM NH4Cl, TBS, washed with TBS, permeabilized for 15 min in 0.1% Triton X-100 in TBS, and washed with TBST (0.05% Tween-20 in TBS). The slides were then blocked for 2 h with 1% BSA(Sigma-Aldrich) in TBST in a humidified chamber at 37 °C and incubated overnight at 4 °C with appropriate combinations of antibodies. After washing with TBST, proximity ligation was performed using the Rabbit PLUS and Mouse MINUS Duolink in situ PLA kits (Sigma-Aldrich) according to the manufacturer’s protocol. Finally, slides were dehydrated, air-dried, and embedded in Citifluor mounting medium (Sigma-Aldrich). Images were recorded by Zeiss LSM700 confocal microscope system using 10 × or 40 × NA APO lens and were analyzed with Zeiss confocal software.

### CRISPRa and CRISPRi

Cells stably expressing dCas9-vp64-Blast (Addgene #61425) or pHR-SFFV-KRAB-dCas9-P2A-mCherry (Addgene #60954) were generated through sequential lentiviral transduction and selection with blasticidin or by fluorescent-activated cell sorting. The resulting cells were then transduced with a lentiviral vector (lentiGuide-puro, Addgene #52963) inserted with gRNA targeting promoter sequences of INHEG and subsequently puromycin-selected to generate the final cell lines stably overexpressing or knockdown INHEG. CHOP-CHOP was used for guide design (http://chopchop.cbu.uib.no/).

### RNA pull-down assay

The RNA pull-down assay was modified from a published procedure^61^. Biotin‐labeled INHEG full‐length (sense), antisense, and control RNA (Lac Z) were obtained with Biotin RNA Labeling Mix (Roche) in vitro. RNA‐binding proteins were pulled down by streptavidin beads. Pull‐down components were separated with SDS–PAGE followed by immunoblotting with anti‐NOP58 or anti-TAF15 antibodies or silver staining. Differential bands enriched by INHEG were analyzed by LTQ Orbitrap XL mass spectrometry. In addition, truncated fragments of INHEG were also in vitro‐transcribed to biotin‐labeled RNA followed by RNA pull‐down and immunoblotting.

### In vitro RNA-protein binding assay with GST-tagged proteins

In vitro RNA-protein binding assay with GST-tagged proteins was performed as previously described with minor modification^20^. Briefly, biotin-labeled full-length INHEG RNA was in vitro transcribed from plasmids containing a T7 promoter by T7 RNA polymerase (Roche), followed by treatment with RNase-free DNase I (Roche) and purified with Trizol (Invitrogen). The mRNAs for NOP56, NOP58, and TAF15 were amplified by RT-PCR and cloned into the pGEX-6P-1 vector (GE Healthcare) and expressed in *Escherichia coli* to purify GST-tagged proteins. INHEG RNA and GST-tagged proteins were co-incubated in binding buffer (20 mM HEPES pH = 7.6, 150 mM KCl, 0.05% NP40, 1 mM DTT, 0.5 mM PMSF) at 4°C for 2 h. The streptavidin beads were then added and incubated with RNA and protein for 1 h. After five times washes with wash buffer (20 mM HEPES pH = 7.6, 300 mM KCl, 0.05% NP40, 1 mM DTT, 0.5 mM PMSF), proteins from the beads were analyzed by western blot with GST antibody.

### Electrophoretic mobility shift assay (EMSA)

In vitro transcribed RNAs were annealed by heating at 65 °C for 5 min, then slowly cooled down to room temperature. RNAs (200 ng) and various amounts of purified His-tagged proteins were incubated in binding buffer (100 mM HEPES pH 7.5, 200 mM KCl, 10 mM MgCl_2_, 10 mM DTT) for 25 min. Binding reactions were then immediately loaded onto 5% nondenaturing polyacrylamide gel. Nucleic acid and protein were stained with an Electrophoretic Mobility Shift Assay (EMSA) Kit (Invitrogen) according to the instructions.

### GST pull-down assay

His-tagged NOP58 expressed by prokaryotes was added to the appropriate GST-tagged TAF15 or GST alone immobilized on glutathione-Sepharose 4B and incubated for 1 h in binding buffer (50 mM Tris-HCl, pH 7.5, 150 mM NaCl, 10 mM MgCl_2_, 10% glycerol, 1% Triton X-100, 1 mM DTT, 1% BSA) at 4 °C. After centrifugation, the pellets were washed five times with binding buffer at 4 °C and detected by western blots.

### Intracranial tumor formation in vivo

Five to six weeks old male and female NSG mice were used in this study. NSG mice were obtained from Shanghai Jihui Laboratory Animal Care Co.,Ltd. Intracranial transplantation of GSCs was performed as previously described^62^. Briefly, GSCs stably overexpressing or knockdown INHEG/FBL/NOP58 were injected into intracranially into the right cerebral cortex of NSG immunocompromised mice. Animals were monitored until neurological signs were observed, at which point they were sacrificed. Neurological signs or signs of morbidity included hunched posture, gait changes, lethargy and weight loss. To compare the tumor growth, brains were isolated from mice implanted with GSCs on the same day when there was development of neurological signs after implantation. Brains were harvested and fixed in 4% formaldehyde for 48 h, embedded in paraffin, and sectioned. H&E staining was performed on sections for histologic analysis. In parallel survival experiments, mice were observed until the development of neurological signs.

### Detection of rRNA methylation by RiboMeth-seq

RiboMeth-seq analyses were performed in DGC and GSC cells based on a previously described protocol^26,63^. In brief, total RNA was dissolved in 50 mM sodium carbonate/bicarbonate buffer (pH 9.2) and heated at 95 °C. Fragmented RNAs were purified with the RNA Clean & Concentrator-5 kit (Zymo Research). Next, Fragmented RNA was 3′-end dephosphorylated and purified with the RNA Clean & Concentrator-5 kit (Zymo Research). RNA was then 5′-end phosphorylated with 20 units of T4 polynucleotide kinase (NEB) and purified again as above. Sequencing libraries were prepared with the VAHTSTM Small RNA Library Prep Kit for Illumina® (Vazyme) following the manufacturer’s instruction and sequencing using the Novaseq-150PE platform.

### RNA-seq

RNA was extracted from cells and tissues using the Trizol total RNA isolation reagent (Invitrogen) according to the manufacturer’s protocol. Sequencing libraries were prepared with the VAHTSTM Total RNA-seq (H/M/R) Library Prep Kit for Illumina (Vazyme) following the manufacturer’s instructions. The libraries were sequenced by the Illumina platform and 150-bp pair-end reads mode.

### Computational analysis for RiboMeth-Seq

Adapters were removed by cutadapt v2.7^64^ with parameters (-a AGATCGGAAGAGCACACGTCTGAACTCCAGTCAC -A GATCGTCGGACTGTAGAACTCTGAAC). Trimmed reads were mapped to ribosome DNA sequence by bowtie v2.2.9^65^ and then sorted by samtools v1.10^66^. BAM files were converted to BED format by bamToBed (bedtools v2.26)^67^. Positions of 5’ (beginnings) and 3’ (ends) were extracted and converted to bedGraph using custom python script. The methylation level was further quantified by MethScore C calculation^68^.

### Computational analysis for RNA-seq

To construct a comprehensive RNA annotation, RNA-seq in our previous study^69^ were aligned to the hg19 reference genome by STAR 2.7.7a^70^ and assembled by cufflinks v2.2.1^71^. The assembled transcriptome was merged with GENCODEv19^72^. Single exon and shorter (<200 bp) transcripts were discarded, and transcripts were further filtered based on coding potential determined by CPC^73^ and CPAT^74^. The final transcriptome was used in the paper. For differential expression analysis, reads were realigned and counted by STAR with the --quantMode GeneCounts option, and differential expressed genes were identified by edgeR^75^.

### Computational analysis for NOP58 RIP-seq

NOP58 RIP-seq data were aligned to the reference genome produced from RNA-seq analysis (see Computational analysis for RNA-seq part) with STAR 2.7.3a and summarized by featureCounts^76^. Enriched genes by NOP58 antibody relative to IgG and input were identified by DESeq2^77^.

### Ribosome profiling

The Ribo-seq was performed as previously described with minor modification^78^. Briefly, INHEG-activated or control patient-derived GSCs (3565) were treated with 100 mg/mL cycloheximide for 5 min at 37 °C before collection. The cells were lysed with polysome lysis buffer (5x Mammalian Polysome Buffer (Epibiotek), 1% Triton X-100, 100 mM DTT, DNase I (1U/μL), 1 mg/mL Cycloheximide, 1% Igepal CA-630). The intact mRNA-ribosome complexes were isolated with Epi™ Ribosome Profiling Kit (Epibiotek) as manual book. The library was sent for sequencing with Illumina NextSeq CN500.

### Bioinformatics analysis for ribosome profiling

Adapter sequences were removed from raw sequencing data using cutadapt software. Meanwhile, reads with length between 25 and 35 bp were kept for downstream analysis. Then reads were aligned to rRNA and tRNA sequences so as to remove rRNA and tRNA reads using bowtie software, remaining reads were used to align to reference genome and transcriptome (Ensembl Version 91) using hisat2 and bowtie software separately. Read counts were calculated using featureCounts software. Raw counts were further normalized as RPKM values using fpkm function in edgeR package. Translational efficiencies were determined as the ratio of (normalized abundance determined by ribosome profiling)/(normalized abundance determined by RNA-seq) as previously repored^79^.

### Reporting summary

Further information on research design is available in the Nature Portfolio Reporting Summary linked to this article.

### Supplementary information

Supplementary Information

Description of Additional Supplementary Files

Supplementary Data 1

Supplementary Data 2

Supplementary Data 3

Reporting Summary

### Source data

Source Data

## Data Availability

The high-throughput sequencing data used in this study are available in the GEO database under accession GSE185695. All other data supporting the findings of this study are available within the paper and its Supplementary Information. Source data are provided with this paper.
