# Supplementary material for: LncRNA INHEG promotes glioma stem cell maintenance and tumorigenicity through regulating rRNA 2’-O-methylation
Source: Nat Commun. 2023 Nov 18;14:7526. doi: 10.1038/s41467-023-43113-5 (PMC10657414; doi:10.1038/s41467-023-43113-5)
Supplement: Supplementary file 1 — Supplementary Information [file 41467_2023_43113_MOESM1_ESM.pdf]

LncRNA INHEG promotes glioma stem cell maintenance and tumorigenicity through regulating rRNA 2'-O methylation

Lihui Liu,1, 11 Ziyang Liu,1, 2, 11 Qinghua Liu,1, 11 Wei Wu,1, 11 Peng Lin,3, 4, 11 Xing Liu,5, 11 Yuechuan Zhang,6 Dongpeng Wang,1 Briana C. Prager,7, 8 Ryan C. Gimple,7 Jichuan Yu,3, 4 Weixi Zhao,3,4 Qiulian Wu,9 Wei Zhang,10 Erzhong Wu,1 Xiaomin Chen,1 Jianjun Luo,1 Jeremy N. Rich,9, * Qi Xie,3,4, * Tao Jiang,5, 10, * Runsheng Chen1, 2, *

Supplementary information

Supplementary Fig. 1

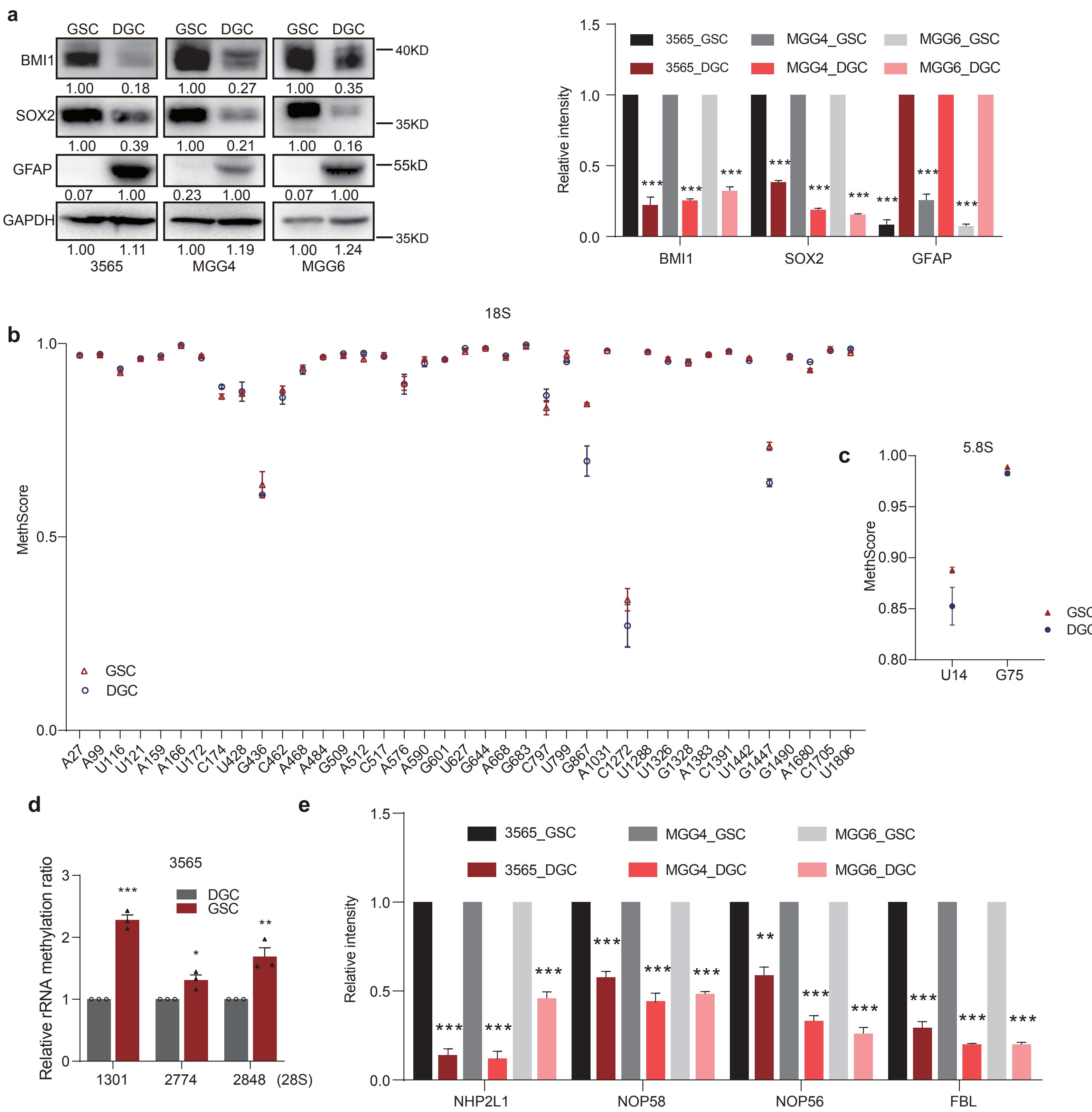

Supplementary Fig. 1| rRNA methylation ratios were higher in patient-derived GSCs than in matched DGCs.

(a) Western blot of BMI1, SOX2, and GFAP in three patient-derived GSCs (3565, MGG4, and MGG6) and matched DGCs. n = 3 independent experiments.

(b and c) MethScore values for 2′-O methylated nucleotide in 18S (A27, A99, U116, U121, A159, A166, U172, C174, U428, G436, C462, A468, A484, G509, A512, C517, A576, A590, G601, U627, G644, A668, G683, C797, U799, G867, A1031, C1272, U1288, U1326, G1328, A1383, C1391, U1442, G1447, G1490, A1680, C1705, U1806) (b) and 5.8S (U14, G75) (c) rRNAs from patient-derived GSCs (3565 and MGG4) and matched DGCs. The MethScore is equal to the ratio of 2′-O-Me at each modified nucleotide. Data represent the mean ± SD from GSCs (3565 and MGG4) and DGCs.

(d) rRNA methylation ratio for sites along 28S rRNA in patient-derived GSCs (3565) and matched differentiated DGCs with RTL-P followed by qPCR. n = 3 independent experiments.

(e) The band densities quantification of western blots in Fig. 1e. n = 3 independent experiments.

Data represent the mean ± SD. *P* values were derived by Student's *t* test. * $P < 0.05$; ** $P < 0.01$; *** $P < 0.001$.

Supplementary Fig. 2

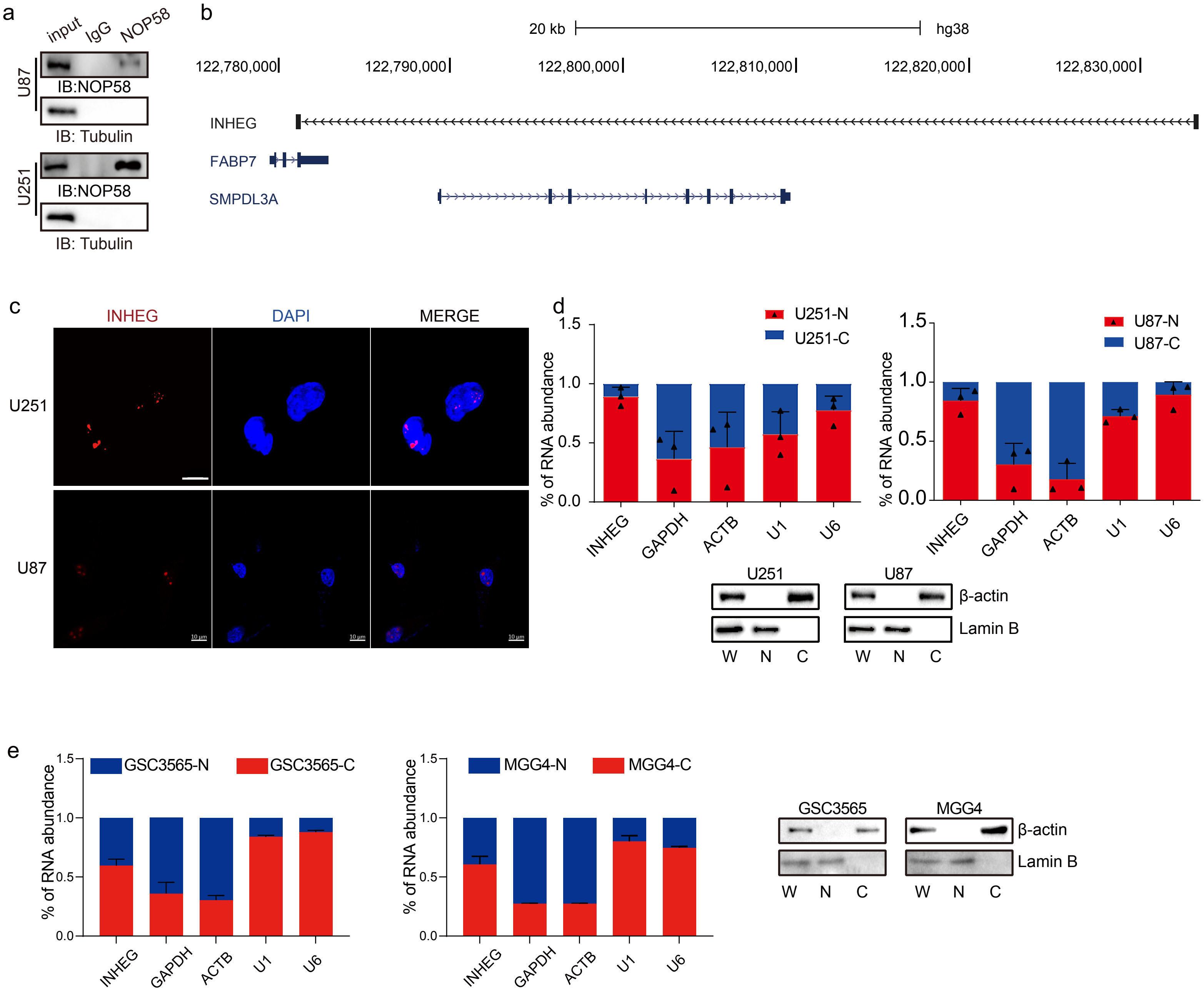

Supplementary Fig. 2| Characterization of novel lncRNA INHEG.

(a) Western blot showing the efficiency and the specificity of UV cross-linked immunoprecipitation of NOP58 in U87-MG and U251-MG cells (representative images from three independent experiments).

(b) Genomic locus of INHEG.

(c) Representative fluorescence images of INHEG (red) detected by RNA fluorescence in situ hybridization in U251-MG and U87-MG cells. Nuclei were stained with DAPI (blue). Scale bar, 10 μm.

(d) Subcellular localization of INHEG analyzed with nucleocytoplasmic fractionation of U87-MG and U251-MG cells followed by qRT-PCR (Top). Nucleocytoplasmic fractionation of U251 and U87 was confirmed by western blot analysis (bottom). W: whole cell lysate; N: nucleus; C: cytoplasm.

(e) Subcellular localization of INHEG analyzed with nucleocytoplasmic fractionation of GSC3565 and MGG4 followed by qRT-PCR and nucleocytoplasmic fractionation confirmed by western blot analysis. W: whole cell lysate; N: nucleus; C: cytoplasm.

(d and e) The data represent the mean ± SD. n = 3..

Supplementary Fig. 3

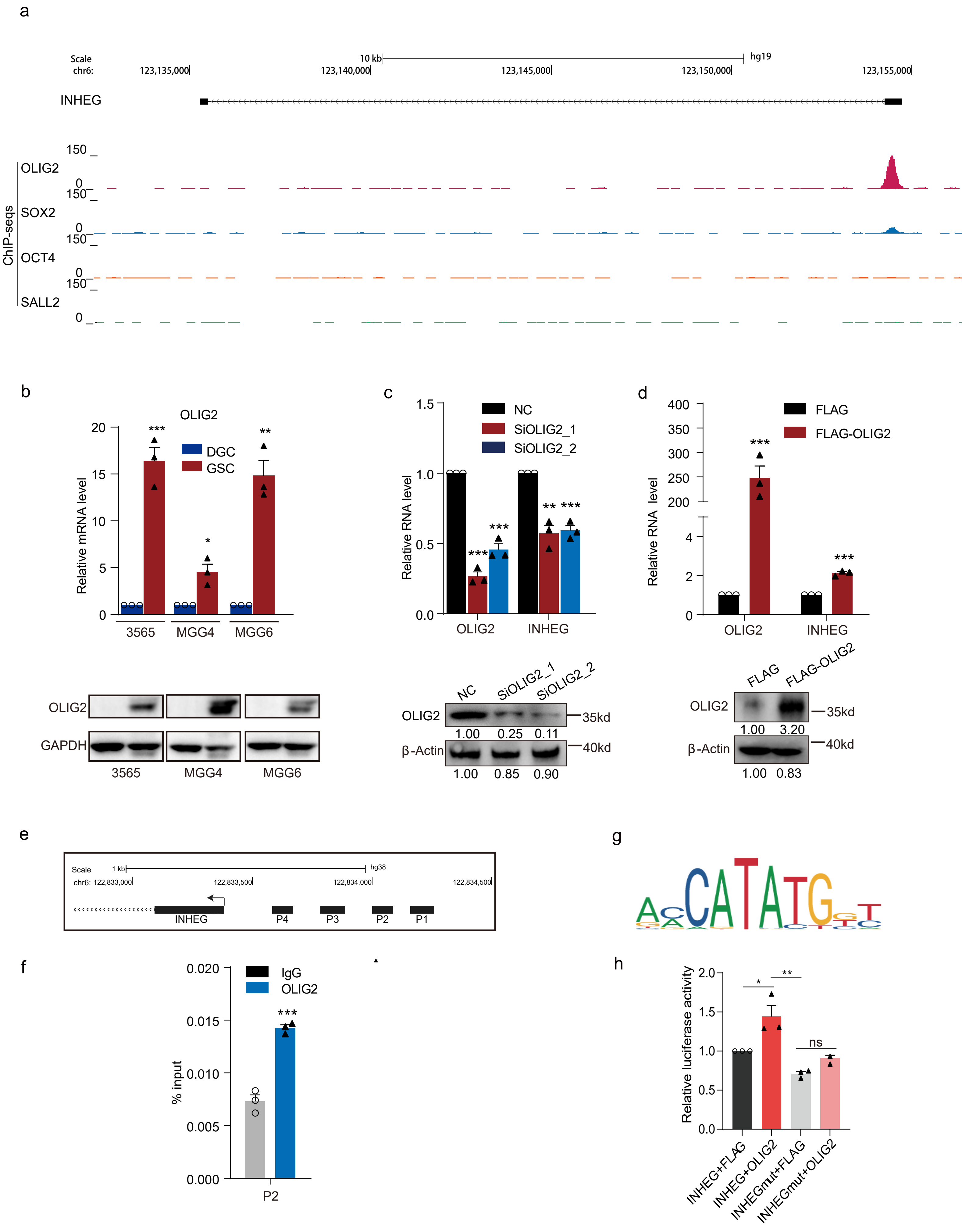

Supplementary Fig. 3| LncRNA INHEG is transcriptionally regulated by OLIG2.

(a) H3K27ac signal at INHEG locus in TPCs induced by POU3F2, SOX2, SALL2, or OLIG2 in public sequencing data.

(b) The relative expression of OLIG2 in patient-derived GSCs and matched DGCs detected by qRT-PCR and western blot.

(c) The relative expression of OLIG2 and INHEG in U251-MG cells treated with control or OLIG2-targeted siRNAs by qRT-PCR and western blot.

(d) The relative expression of OLIG2 and INHEG in U251-MG cells treated with control or OLIG2-overexpressed plasmids by qRT-PCR and western blot.

(e) Genomic locus of INHEG promoter.

(f) The ChIP assay of binding of OLIG2 to INHEG promoter DNA in U251-MG.

(g) The consensus OLIG2 binding site in the promoter region of INHEG by FIMO searching.

(h) Luciferase reporter assays for 293T cells transfected with reporter plasmids containing wildtype or mutated INHEG promoters as well as control or OLIG2-overexpressing plasmids.

Data are shown as mean ± SD. n = 3 independent experiments. Significance determined by two tailed Student’s *t*-test. *$p < 0.05$; **$p < 0.01$; ***$p < 0.001$.

Supplementary Fig. 4

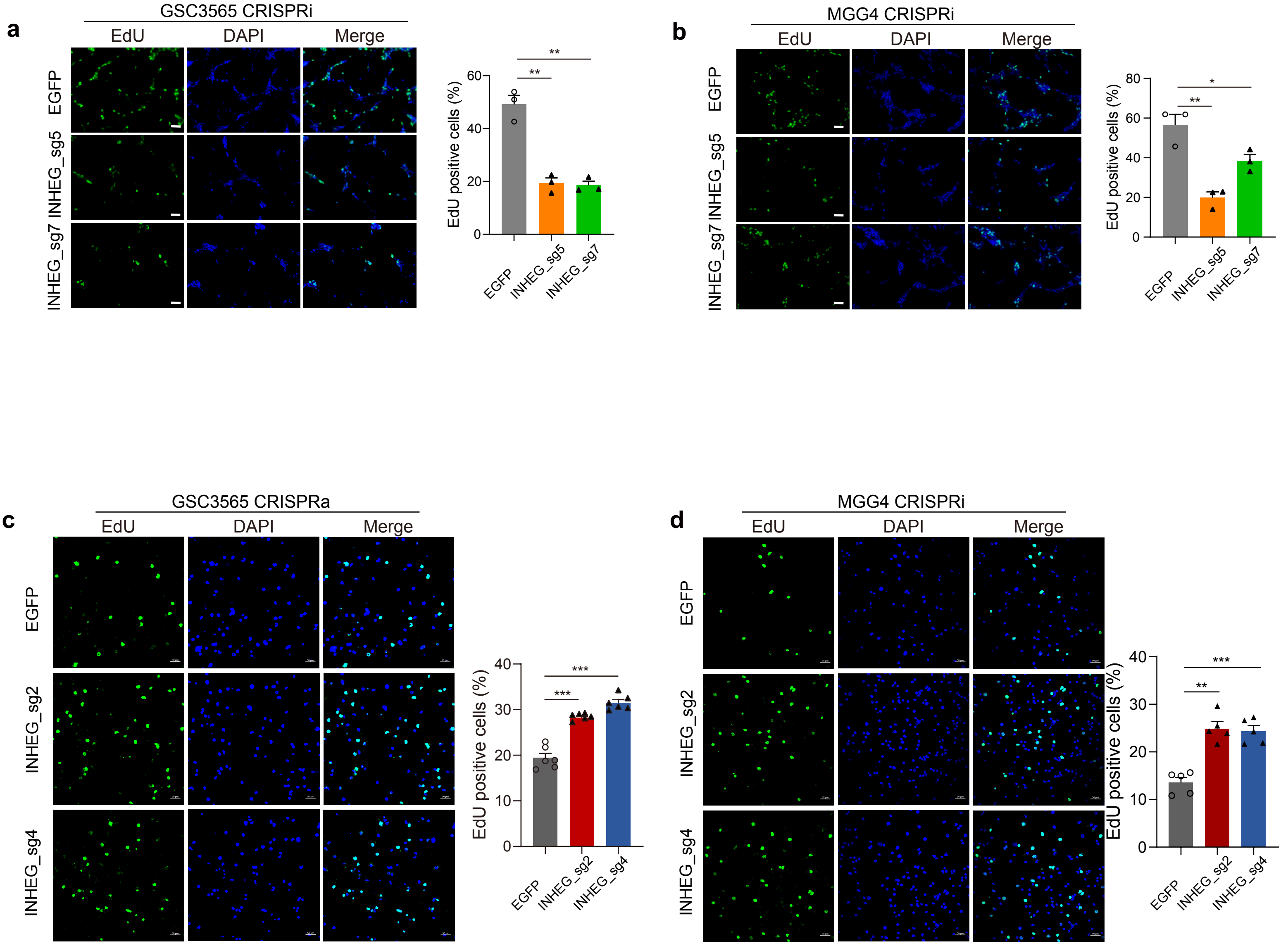

Supplementary Fig. 4| EdU proliferation assay analysis of the effect of INHEG on the growth of GSCs.

(a and b) EdU proliferation assays for GSC3565(a) and MGG4(b) following KRAB-dCas9 mediated INHEG knockdown or treatment with a non-targeting sgRNA. Left: representative images of cells. Proliferating cells that have incorporated EdU are stained red, and nuclei were stained with DAPI (blue). Scale bar: 50 μm. Right: statistical analysis of EdU positive cells.

(c and d) EdU proliferation assays for GSC3565(c) and MGG4(d) following dCas9-VP64 mediated INHEG overexpression or treatment with a non-targeting sgRNA. Left: representative images of cells. Proliferating cells that have incorporated EdU are stained red, and nuclei were stained with DAPI (blue). Scale bar: 50 μm. Right: statistical analysis of EdU positive cells.

Data are shown as mean ± SD. $n = 3$ (a and b) or 5 (c and d) independent experiments. Significance determined by two tailed Student's t-test. *$p < 0.05$; **$p < 0.01$; ***$p < 0.001$.

Supplementary Fig. 5

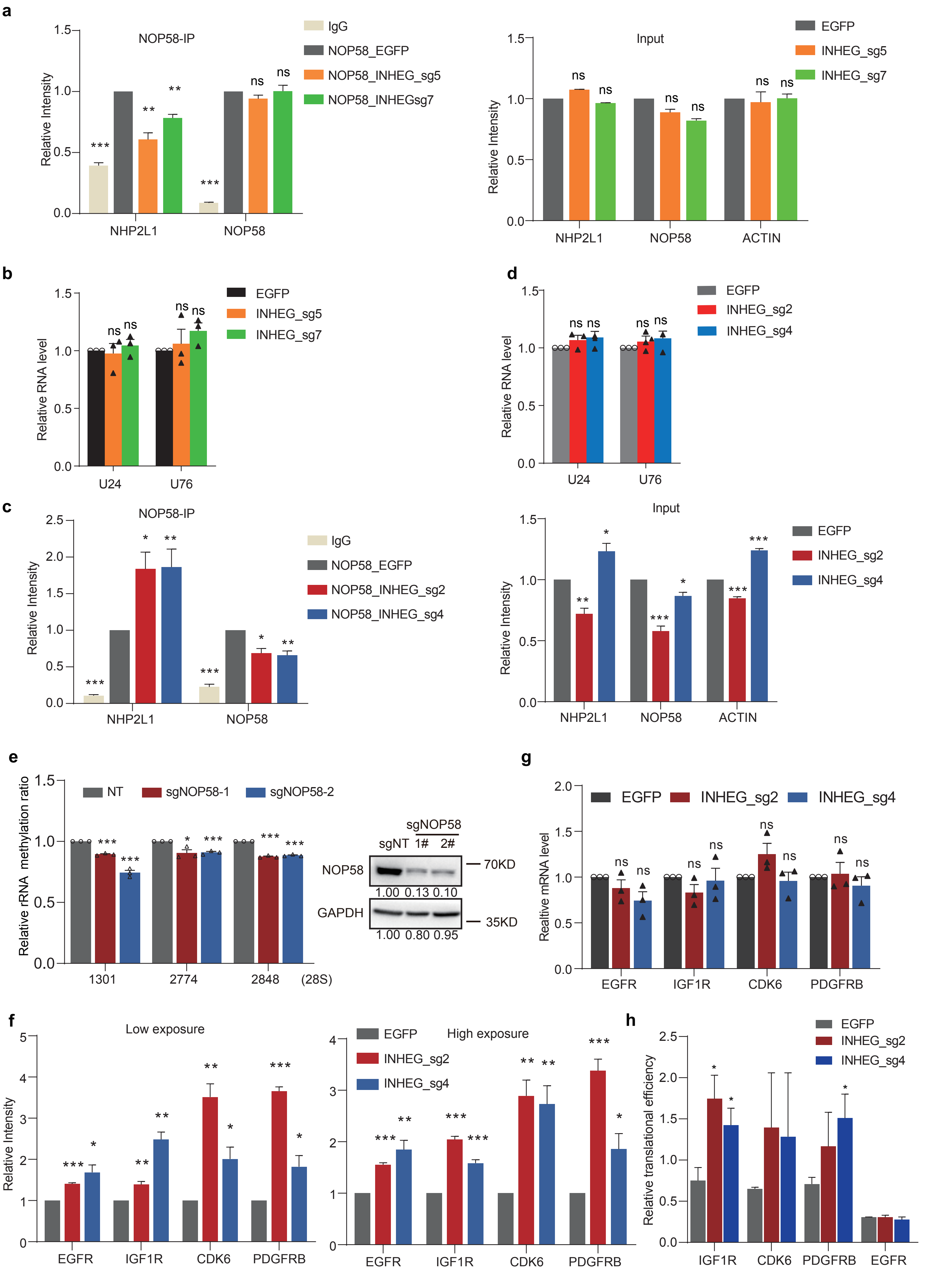

Supplementary Fig. 5| INHEG and NOP58 modulate rRNA 2’-O-methylation status.

(a) The band densities quantification of western blots in Fig. 5a. $n = 3$ independent experiments.

(b) The expression of U24 or U76 in U251-MG cells following KRAB-dCas9 mediated INHEG knockdown or treatment with a non-targeting sgRNA.

(c) The band densities quantification of western blots in Fig. 5c. $n = 3$ independent experiments.

(d) The expression of U24 or U76 in U251-MG cells following dCas9-VP64 mediated INHEG overexpression or treatment with a non-targeting sgRNA.

(e) rRNA methylation ratio for sites along 28S rRNA in GSC3565 after treatment with Cas9 and a non-targeting control or two NOP58-targeting sgRNAs.

(f) The band densities quantification of western blots in Fig. 5i.

(g) The qRT-PCR analysis of EGFR, IGF1R, CDK6 and PDGFRB mRNA in GSC3565 following dCas9-VP64 mediated INHEG overexpression or treatment with a non-targeting sgRNA.

(h) The relative translational efficiencies (TE) of IGF1R, CDK6, PDGFRB, and EGFR mRNA detected by RNA-seq and Ribo-seq in GSC3565 cells after treatment with Cas9 and non-targeting control or INHEG-targeting sgRNAs.

Data are shown as mean ± SD. $n = 3$ biological replicates. Significance determined by two tailed Student’s *t*-test. *p < 0.05; **p < 0.01; ***p < 0.001; ns, no significance.

Supplementary Fig. 6

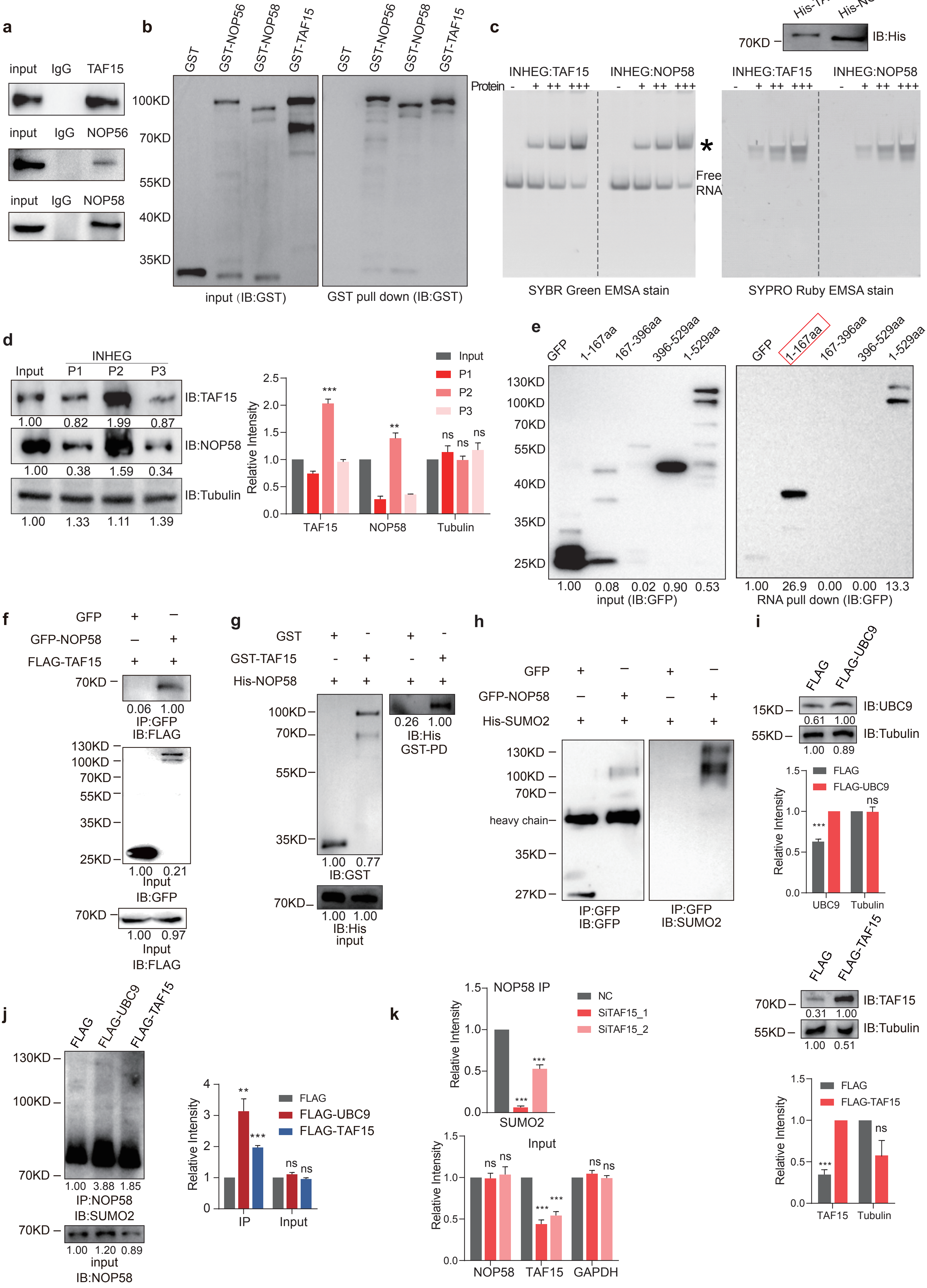

Supplementary Fig. 6| TAF15 interacts with NOP58 and regulates NOP58 sumoylation.

(a) Western blot of immunoprecipitation of TAF15, NOP56 and NOP58.

(b) GST pull-down assay of the binding of INHEG to GST-tagged TAF15, NOP56 or NOP58.

(c) Electrophoretic mobility shift assay of the binding of INHEG to His-tagged TAF15 or NOP58. The shift bands were marked by the asterisk. Increasing amounts of TAF15 or NOP58 protein were added to INHEG binding reaction. Left: The gel stained with SYBR Green EMSA stain. Middle: The gel stained with SYPRO Ruby EMSA stain. Right: The prokaryotically-expressed His-tagged proteins analyzed by western blot (representative images from three independent experiments).

(d) RNA pull-down assay of the binding of truncated INHEG to TAF15 and NOP58.

(e) RNA pull-down assay of the binding of INHEG to truncated NOP58.

(f) Co-precipitation assay of the binding of FLAG-TAF15 to GFP-NOP58 with GFP antibody in whole-cell lysates of TAF15- and NOP58- ectopically expressed 293T cells.

(g) GST pull-down assay of the binding of prokaryotically expressed recombinant human His-tagged NOP58 to GST-tagged TAF15.

(h) Exogenous NOP58 sumoylation detection in NOP58- and SUMO2-ectopically expressed 293T cells.

(i) The relative expression of UBC9 (top) and TAF15 (bottom) in control and UBC9 or TAF15-overexpressed U87-MG cells.

(j) Endogeneous NOP58 sumoylation detection in control or UBC9/TAF15 ectopically expressed U87-MG cells.

(k) The band densities quantification of western blots in Fig. 6j.

(a, b, e, f, g, and h) Representative images from three independent experiments. (d, I, j, and k) The histograms represent the mean ± SD. n = 3 independent experiments. P values were derived by Student's t test. * P < 0.05;** P < 0.01; *** P < 0.001.

Supplementary Fig. 7

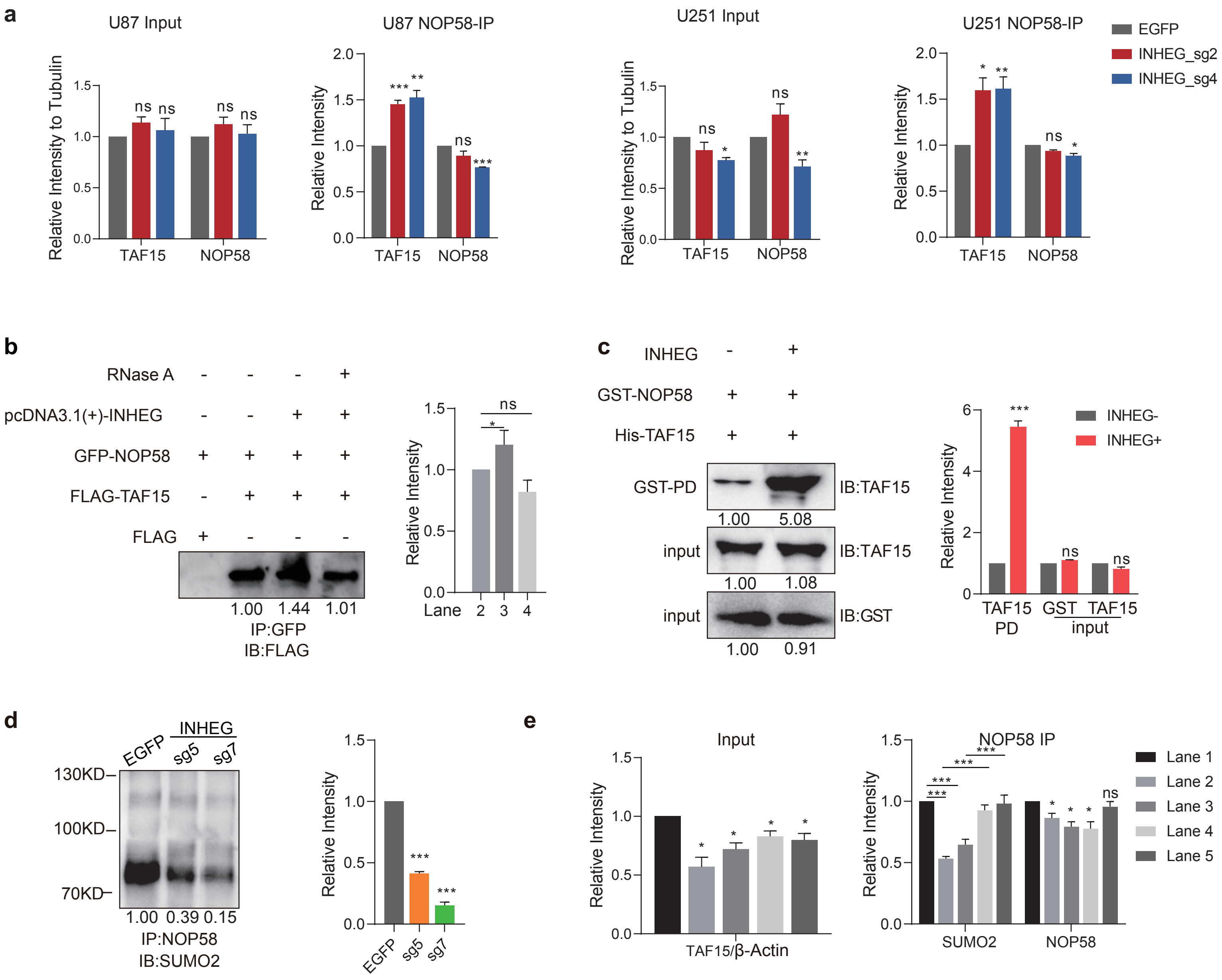

Supplementary Fig. 7| INHEG enhances the interaction between TAF15 and NOP58 and regulates NOP58 sumoylation.

(a) The band densities quantification of western blots in Fig. 6k.

(b) Exogenous co-immunoprecipitation of TAF15 and NOP58 after ectopic expression of INHEG in 293T cell lysate treated with RNase A or not.

(c) GST pull-down assay of the binding His-tagged NOP58 to GST-tagged TAF15 with INHEG added or not.

(d) Endogeneous NOP58 sumoylation detection in U251-MG cells following KRAB-dCas9 mediated INHEG knockdown or treatment with a non-targeting sgRNA.

(e) The band densities quantification of western blots in Fig. 6m.

The histograms represent the mean ± SD. *n* = 3 independent experiments. P values were derived by Student's t test. * $P < 0.05$; ** $P < 0.01$; *** $P < 0.001$.

Supplementary Fig. 8

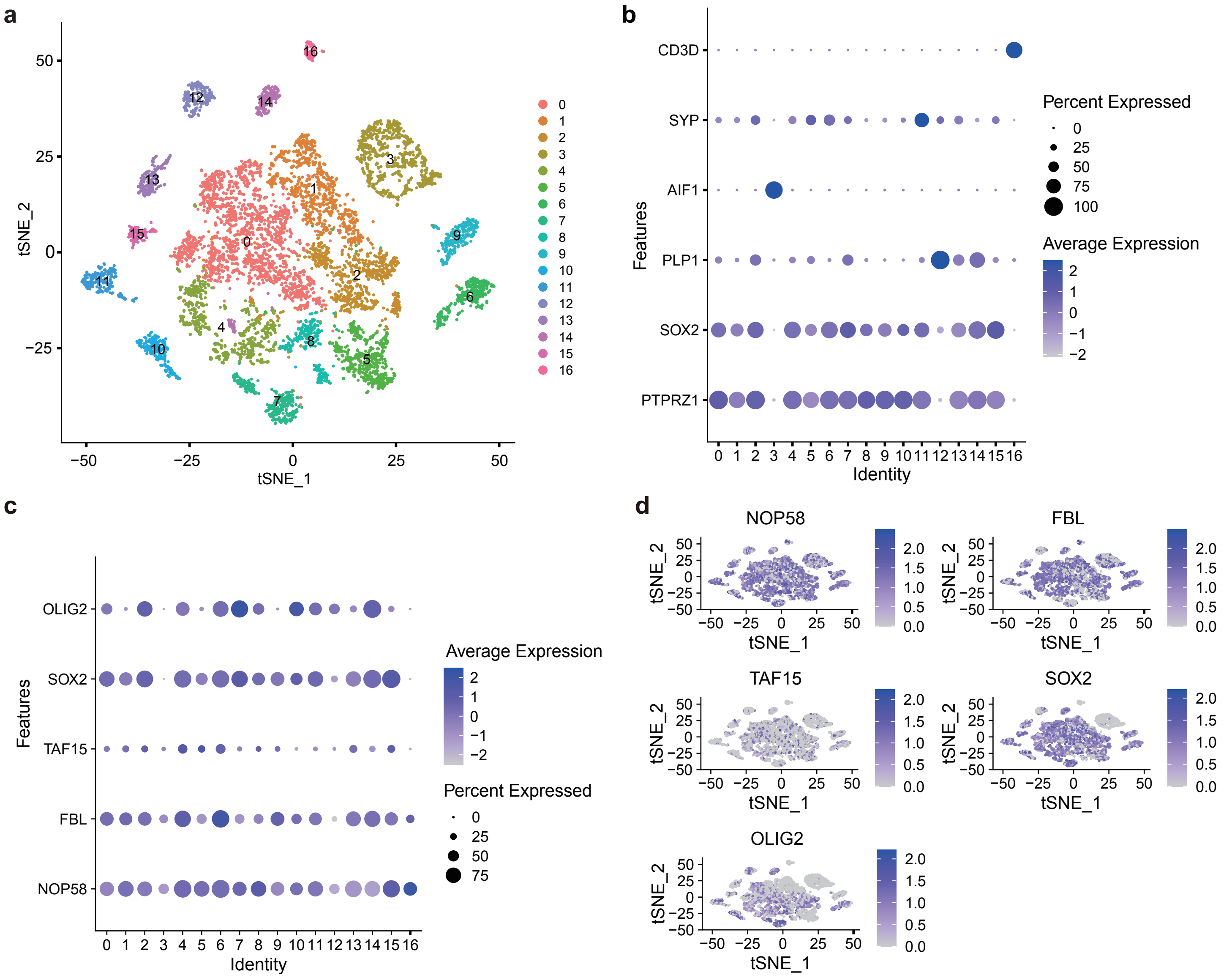

Supplementary Fig. 8| NOP58 and FBL were significantly co-express with glioma stem cell markers, SOX2 and OLIG2.

(a) t-distributed stochastic neighbour embedding (t-SNE) plot of 17 cell clusters annotated in scRNA-seq data.

(b) Dot plots showing the expression of selected signature genes in each cluster.

(c) Dot plots showing the expressions of OLIG2, SOX2, TAF15, FBL, and NOP58 in each cluster.

(d) t-SNE plot showing the expressions of OLIG2, SOX2, TAF15, FBL, and NOP58 in each cluster.

Supplementary Fig. 9

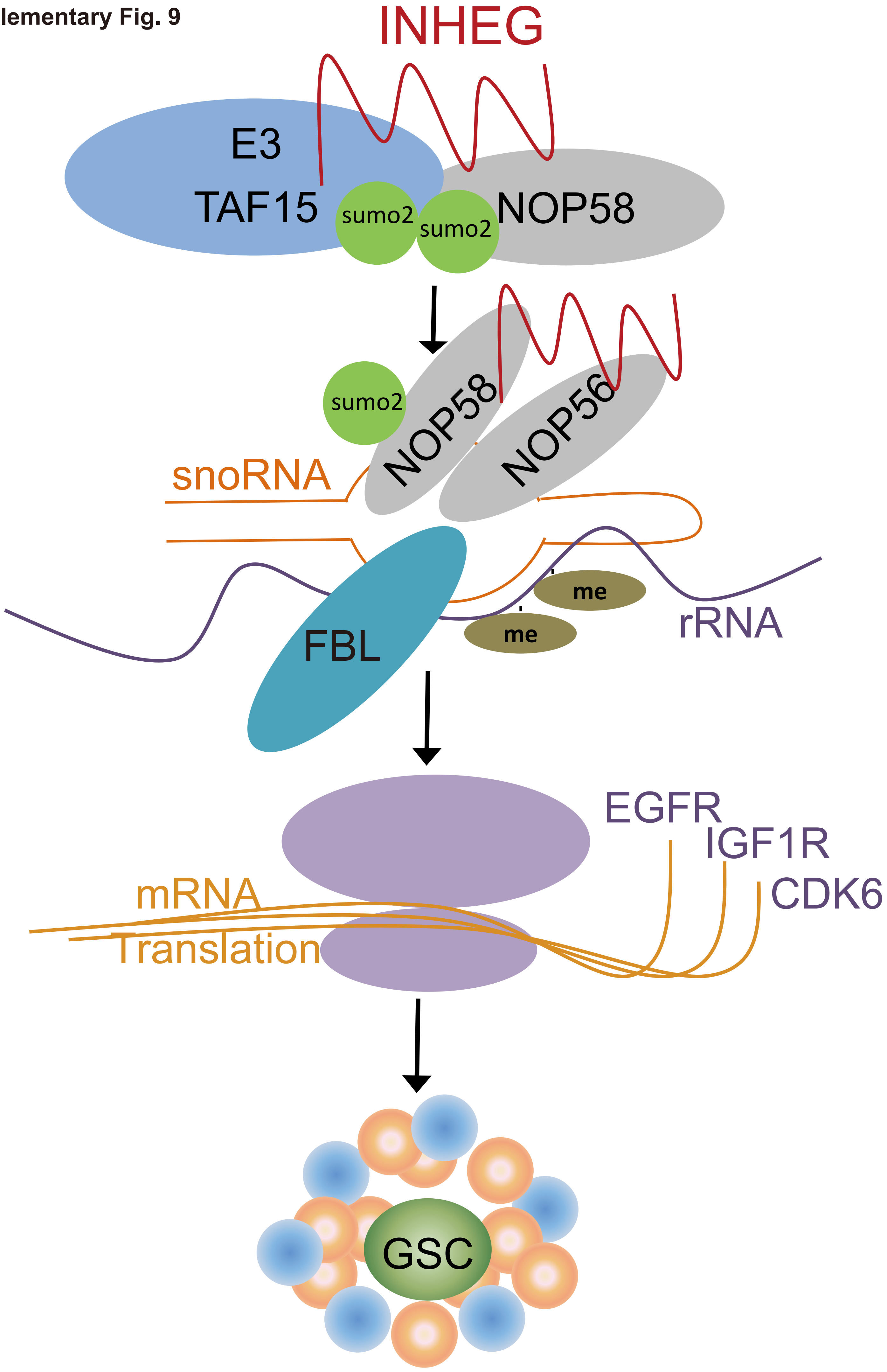

Supplementary Fig. 9| Working model of INHEG promoting GSC self-renewal.

Supplementary Table 1. RNA pull-down-MS analysis showing the enriched proteins by lncRNA INHEG.

Protein	PSMs_sense	PSMs_antisense
NOP58	9	6
VIM	9	7
NOP56	13	7
GNL3	6	3
RPL19	4	2
RNH1	3	5
GPATCH4	6	1
TCOF1	4	0
RPS6	7	6
KNOP1	3	1
ZFP37	3	1
PGAM5	2	1
DCD	2	0
NKAP	2	0
PTRF	3	2
RPL24	2	0
RPS23	2	2
RPL27A	2	1
ZCCHC17	3	0
RRP1B	3	2
RPS3A	2	0
TAF15	2	0

Supplementary Table 1. Transcriptome analysis showing the differentially expressed genes in control and NOP58/FBL knockdown GSCs (3565 and MGG4).

Supplementary Table 2. The uvRIP-seq analysis showing the enriched RNAs by NOP58.

Supplementary Table 3. RNA pull-down-MS analysis showing the enriched proteins by lncRNA INHEG.

Supplementary Table 4. The primers used for real-time-qPCR or reverse transcription-PCR analysis.
