# Supplementary material for: LncRNA INHEG promotes glioma stem cell maintenance and tumorigenicity through regulating rRNA 2’-O-methylation
Source: Nat Commun. 2023 Nov 18;14:7526. doi: 10.1038/s41467-023-43113-5 (PMC10657414; doi:10.1038/s41467-023-43113-5)
Supplement: Supplementary file 2 — Description of Additional Supplementary Files [file 41467_2023_43113_MOESM2_ESM.pdf]

Description of Additional Supplementary Files

Supplementary Data 1. Transcriptome analysis showing the differentially expressed genes in control and NOP58/FBL knockdown GSCs (3565 and MGG4).

Supplementary Data 2. The uvRIPseq analysis showing the enriched RNAs by NOP58.

Supplementary Data 3. The primers used for real-time-qPCR or reverse transcription-PCR analysis.
