# Supplementary material for: LncRNA INHEG promotes glioma stem cell maintenance and tumorigenicity through regulating rRNA 2’-O-methylation
Source: Nat Commun. 2023 Nov 18;14:7526. doi: 10.1038/s41467-023-43113-5 (PMC10657414; doi:10.1038/s41467-023-43113-5)
Supplement: Supplementary file 6 — Reporting Summary [file 41467_2023_43113_MOESM6_ESM.pdf]

nature portfolio

Corresponding author(s): Runsheng Chen, Tao Jiang, Qi Xie, Jeremy N. Rich

Last updated by author(s): Sep 28, 2023

Reporting Summary

Nature Portfolio wishes to improve the reproducibility of the work that we publish. This form provides structure for consistency and transparency in reporting. For further information on Nature Portfolio policies, see our Editorial Policies and the Editorial Policy Checklist.

Statistics

For all statistical analyses, confirm that the following items are present in the figure legend, table legend, main text, or Methods section.

n/a	Confirmed	
☐	☒	The exact sample size (*n*) for each experimental group/condition, given as a discrete number and unit of measurement
☐	☒	A statement on whether measurements were taken from distinct samples or whether the same sample was measured repeatedly
☐	☒	The statistical test(s) used AND whether they are one- or two-sided<br>*Only common tests should be described solely by name; describe more complex techniques in the Methods section.*
☒	☐	A description of all covariates tested
☒	☐	A description of any assumptions or corrections, such as tests of normality and adjustment for multiple comparisons
☐	☒	A full description of the statistical parameters including central tendency (e.g. means) or other basic estimates (e.g. regression coefficient) AND variation (e.g. standard deviation) or associated estimates of uncertainty (e.g. confidence intervals)
☐	☒	For null hypothesis testing, the test statistic (e.g. *F*, *t*, *r*) with confidence intervals, effect sizes, degrees of freedom and *P* value noted<br>*Give P values as exact values whenever suitable.*
☒	☐	For Bayesian analysis, information on the choice of priors and Markov chain Monte Carlo settings
☒	☐	For hierarchical and complex designs, identification of the appropriate level for tests and full reporting of outcomes
☒	☐	Estimates of effect sizes (e.g. Cohen's *d*, Pearson's *r*), indicating how they were calculated

Our web collection on statistics for biologists contains articles on many of the points above.

Software and code

Policy information about availability of computer code

Data collection

The RNA-seq data was sequenced by Illumina NovaSeq 6000 in PE mode. The uvRIP-seq data was sequenced by Illumina HiSeq X ten in PE mode. The RiboMeth-seq data was sequenced by Illumina NovaSeq-150PE. Ribo-seq data was sequenced by Illumina NextSeq CN500.

Data analysis

Sequencing data was analyzed as described in the Material and methods section.
The following public softwares were used for sequencing data analysis:
STAR 2.7.7a
cufflinks v2.2.1
samtools v1.10
bowtie v2.2.9
bedtools v2.26
cutadapt v2.7
R v3.6.3
subread v2.0.0
DESeq2 v1.26.0
edgeR v3.28.1
hisat2
Ordinary ANOVA and T-test analysis were done with GraphPad Prism 9.

For manuscripts utilizing custom algorithms or software that are central to the research but not yet described in published literature, software must be made available to editors and reviewers. We strongly encourage code deposition in a community repository (e.g. GitHub). See the Nature Portfolio guidelines for submitting code & software for further information.

Data

Policy information about availability of data

All manuscripts must include a data availability statement. This statement should provide the following information, where applicable:

- Accession codes, unique identifiers, or web links for publicly available datasets
- A description of any restrictions on data availability
- For clinical datasets or third party data, please ensure that the statement adheres to our policy

The high-throughput sequencing data used in this study are available in the GEO database under accession GSE185695. All other data supporting the findings of this study are available within the paper as well as its Supplementary Information and Supplementary Data. Source data are provided with this paper.

Human research participants

Policy information about studies involving human research participants and Sex and Gender in Research.

Reporting on sex and gender	The study did not involve sex or gender-based analysis.
Population characteristics	1. Peripheral brain tissue, male, 24 years-old, left frontal, temporal and insular lobes, astrocytoma, IDH-mutant, CNS WHO grade 3<br>2. Peripheral brain tissue, male, 53 years-old, right frontal and insular lobes, astrocytoma, IDH-mutant, CNS WHO grade 2<br>3. Peripheral brain tissue, male, 30 years-old, left frontal lobe, astrocytoma, IDH-mutant, CNS WHO grade 2<br>4. Tumor tissue, male, 49 years-old, left temporal and parietal lobes, glioblastoma, IDH-wildtype, CNS WHO grade 4<br>5. Tumor tissue, male, 31 years-old, left frontal and parietal lobes, glioblastoma, IDH-mutant, CNS WHO grade 4<br>6. Tumor tissue, female, 56 years-old, left frontal, temporal and insular lobes, glioblastoma, IDH-wildtype, CNS WHO grade 4<br>7. Tumor tissue, male, 65 years-old, left parietal lobe, glioblastoma, IDH-wildtype, CNS WHO grade 4<br>8. Tumor tissue, female, 60 years-old, right temporal, parietal, and insular lobes, glioblastoma, IDH-wildtype, CNS WHO grade 4<br>9. Tumor tissue, female, 64 years-old, right frontal lobe, glioblastoma, IDH-wildtype, CNS WHO grade 4.<br>10. Tumor tissue, male, 44 years-old, right frontal and temporal lobe, glioblastoma, IDH-wildtype, CNS WHO grade 4<br>11. Tumor tissue, male, 67 years-old, left temporal lobe, glioblastoma, IDH-wildtype, CNS WHO grade 4
Recruitment	The informed consent was obtained from patients who received a craniotomy operation at Beijing Tiantan Hospital. The surgical resected specimens were then subjected to RNA extraction. Patients were selected according to the availability and quality of tumor material before start of treatment and at acquired resistance. There is no potential self-selection bias.
Ethics oversight	Ethical approval for this study was obtained from the institutional review board of Beijing Tiantan Hospital.

Note that full information on the approval of the study protocol must also be provided in the manuscript.

Field-specific reporting

Please select the one below that is the best fit for your research. If you are not sure, read the appropriate sections before making your selection.

☒ Life sciences ☐ Behavioural & social sciences ☐ Ecological, evolutionary & environmental sciences

For a reference copy of the document with all sections, see nature.com/documents/nr-reporting-summary-flat.pdf

Life sciences study design

All studies must disclose on these points even when the disclosure is negative.

Sample size	The sample size was determined acco rding to the reports in the related research subjects. In vivo mice experiments, we used at least 5 mice per group which is sufficient to detect meaningful biological difference and followed the 3 R's of animal research. No statistical methods were used to predetermine sample sizes.
Data exclusions	No data points were excluded from data ananlysis.
Replication	As reported in the figure legends or Methods section, the findings were reliably reproduced with similar results. For example, in vivo and in vitro experiments involved at least two distinct cell lines. Two separate sgRNAs were used in the knockdown experiments. The in vitro assays were repeated at least three times independently. All the attempt for replication were successful.
Randomization	All mice were randomly allocated into experimental groups. For in vitro experiments, all samples were analyzed equally with no sub-sampling, and therefore there was no requirement for randomization.

Blinding | The investigators were blinded to group allocation during data collection and analysis.

Reporting for specific materials, systems and methods

We require information from authors about some types of materials, experimental systems and methods used in many studies. Here, indicate whether each material, system or method listed is relevant to your study. If you are not sure if a list item applies to your research, read the appropriate section before selecting a response.

Materials & experimental systems

n/a	Involved in the study	
☐	☒	Antibodies
☐	☒	Eukaryotic cell lines
☒	☐	Palaeontology and archaeology
☐	☒	Animals and other organisms
☒	☐	Clinical data
☒	☐	Dual use research of concern

Methods

n/a	Involved in the study	
☒	☐	ChIP-seq
☒	☐	Flow cytometry
☒	☐	MRI-based neuroimaging

Antibodies

Antibodies used

Primary antibodies:
TAF15 Monoclonal Antibody (Invitrogen, Cat# MA3-078, clone 8TA-2B10);
Rabbit anti-TAFII68 Antibody (BETHYL, Cat# A300-307A);
Rabbit anti-NOP56 Antibody (BETHYL, Cat# A302-720A);
NOP58 Rabbit pAb (ABclonal Cat# A4749; RRID:AB_2765846);
Anti-NOP58 Antibody (abcam Cat# ab155969);
SUMO2/3 Rabbit pAb (ABclonal Cat# A2571; RRID:AB_2764457);
SUMO1 Polyclonal Antibody (Proteintech, Cat# 10329-1-AP);
SUMO-2/3 (18H8) Rabbit mAb (Cell Signaling Technology, Cat# 4971S);
UBC9 Polyclonal Antibody (Proteintech, Cat# 10070-1-AP);
Anti-NHP2L1 Antibody (abcam, Cat# ab181982);
Anti-Fibrillarin Antibody (abcam, Cat# ab4566);
SOX2 Polyclonal Antibody (Proteintech, Cat# 11064-1-AP);
OLIG2 Polyclonal Antibody (Proteintech, Cat# 13999-1-AP);
NANOG Polyclonal Antibody (Proteintech, Cat# 14295-1-AP);
CD133 Polyclonal Antibody (Proteintech, Cat# 18470-1-AP);
SUMO2/3 Polyclonal Antibody (Proteintech, Cat# 11251-1-AP);
Beta Actin Monoclonal Antibody (Proteintech, clone2D4H5, Cat# 66009-1-Ig);
GAPDH Monoclonal Antibody (Proteintech, clone 1E6D9, Cat# 60004-1-Ig);
6*His, His-Tag Monoclonal Antibody (Proteintech, clone 1B7G5, Cat# 66005-1-Ig);
DYKDDDDK Tag Polyclonal Antibody (Proteintech, Cat# 20543-1-AP);
GFP Tag Polyclonal Antibody (Proteintech, Cat# 50430-2-AP);
Mouse anti GFP-Tag mAb (ABclonal, Cat# AE012, RRID: AB_2770402);
Anti-GST Antibody (abcam, Cat# ab111947);
Anti-EGFR Antibody (abcam, Cat# ab52894);
IGF1R Rabbit pAb (ABclonal, Cat# A0243, RRID: AB_2757056);
Anti-alpha Tubulin Antibody (abcam, Cat# ab7291)
Lamin B1 Polyclonal Antibody (Proteintech, Cat# 12987-1-AP)
Cdk6 Polyclonal Antibody (Immunoway, Cat# YT5884)
PDGFR-β Polyclonal Antibody (Immunoway, Cat# YT3639)
GFAP Polyclonal Antibody (Proteintech, Cat# 16825-1-AP)
BMI1 Polyclonal Antibody (Proteintech, Cat# 10832-1-AP)

Secondary antibodies:
Goat Anti-Rabbit IgG H&L (HRP) (abcam, Cat# ab6721);
Anti-mouse IgG, HRP-linked Antibody (Cell Signaling Technology, Cat# 7076S);
Goat anti-Rabbit IgG (H+L) Highly Cross-Adsorbed
Secondary Antibody, Alexa Fluor Plus 488 (Invitrogen, Cat# A32731);
Goat anti-Mouse IgG (H+L) Cross-Adsorbed
Ready Probes Secondary Antibody, Alexa Fluor 594 (Invitrogen, Cat# R37121);
Goat anti-Rabbit IgG (H+L) Cross-Adsorbed
Ready Probes Secondary Antibody, Alexa Fluor 594 (Invitrogen, Cat# R37117);

Validation	All antibodies used in this study were validated by manufacturers for that specific application. Relevant validating results can be found in the website of each manufacturer. All antibody stainings were done with negative controls to guarantee specificity. For those antibodies for immunoblots, specificity was validated by detection of the correct molecular weight. All antibodies can be linked to other publications.

Eukaryotic cell lines

Policy information about cell lines and Sex and Gender in Research

Cell line source(s)	HEK293T cells (CRL-3216) , U251 and U87 glioma cells were obtained from American Type Culture Collection (ATCC). MGG4 and MGG6 GSCs lines were from Laboratory of Hiroaki Wakimoto in Massachusetts General Hospital. GSC3565 lines were derived from human (female) primary GBM in our labs. All these cell lines were listed in the manuscript.
Authentication	The authentication of HEK293T cells was provided by commercial companies.
Mycoplasma contamination	All cells used in this study were confirmed to be free from mycoplasma by using TransDetect PCR Mycoplasma Detection Kit (Transgen, FM311-01).
Commonly misidentified lines (See ICLAC register)	No commonly misidentified cell lines were used.

Animals and other research organisms

Policy information about studies involving animals; ARRIVE guidelines recommended for reporting animal research, and Sex and Gender in Research

Laboratory animals	Five to six weeks old male and female NSG mice were used in this study. NSG mice were obtained from Shanghai Jihui Laboratory Animal Care Co.,Ltd. Animals were housed in Westlake University animal center, in temperature between 20-24 ℃, 40%-70% humidity and a 12h light/dark cycle.
Wild animals	The study did not involve wild animals.
Reporting on sex	The study did not involve sex-based analysis.
Field-collected samples	The study did not involve filed-collected samples.
Ethics oversight	All mice procedures were performed under an animal protocol approved by the Institutional Animal Care and Use Committee guidelines of Westlake University.

Note that full information on the approval of the study protocol must also be provided in the manuscript.
